# Survival strategies of mycoplasmas: the critical role of post-translational modifications

**DOI:** 10.3389/fcimb.2025.1688880

**Published:** 2025-11-26

**Authors:** Tingting Li, Hongxia Yuan, Wenjun Zhang, Fangyi Guo

**Affiliations:** 1 Changde Hospital, Xiangya School of Medicine, Central South University (The First People’s Hospital of Changde City), Changde, China; 2 The First People’s Hospital of Chenzhou, First Clinical College, Xiangnan University, Chenzhou, China

**Keywords:** mycoplasmas, post-translational modifications, phosphorylation, acetylation, glycosylation

## Abstract

Mycoplasmas are unique prokaryotic pathogens distinguished by their lack of a cell wall. These microorganisms are widespread in nature and can cause severe infections, leading to substantial tissue damage. Recent advances in mycoplasmology, driven by developments in molecular biology and proteomics, have provided novel insights into their pathogenicity and pathogenic mechanisms. However, critical knowledge gaps remain in understanding their biology. Emerging evidence highlights the crucial role of protein post-translational modifications (PTMs) in regulating mycoplasma physiology, including virulence, metabolic adaptation, and persistence. Investigating mycoplasma PTMs in greater depth promises to expand our understanding of their pathogenic strategies and may reveal new targets for therapeutic intervention against mycoplasma-associated diseases.

## 1 Introduction

Mycoplasmas are among the smallest prokaryotic microorganisms capable of autonomous growth in cell-free media (Dallo and Baseman, 2000). Unlike most bacteria, they lack a cell wall and instead possess a typical lipid bilayer membrane structure. This membrane, like other biological membranes, appears trilaminar in electron microscopy—with two electron-dense layers containing phospholipid head groups and surface proteins, and a more lucent central layer containing acyl groups and cholesterol (Principi and Esposito, 2001). This structural simplicity contributes to their pleomorphic morphology, exhibiting spherical, rod-shaped, and filamentous forms. Mycoplasmas have a highly reduced genome (5.8×10^5^-2.2×10^6^ base pairs) with a G+C content of 23-40%, resembling that of Gram-positive bacteria (Dybvig and Voelker, 1996). Despite their genomic minimalism, they retain essential biological functions, including proliferation, genetic inheritance, and mutation (Macek et al., 2019; Li et al., 2020), many of which are regulated by post-translational modifications (PTMs).

Among the numerous mycoplasma species, only five are currently recognized as pathogens in immunocompetent individuals: *Mycoplasma pneumoniae* (*M. pneumoniae*), *Mycoplasma genitalium* (*M. genitalium*), *Mycoplasma hominis*, *Ureaplasma urealyticum* (*U. urealyticum*), and *Ureaplasma parvum*, while other mollicutes such as *Mycoplasma fermentans* and *Mycoplasma penetrans* can cause infections in immunodeficient patients (Blanchard and Montagnier, 1994; Choppa et al., 1998; Huang et al., 2015). *M. pneumoniae*, for instance, is a leading cause of community-acquired pneumonia and respiratory tract infections (Hammerschlag, 2001), while *U. urealyticum* and *M. genitalium* are associated with non-gonococcal urethritis and pelvic inflammatory disease (Guo et al., 2022; Tuddenham et al., 2022). In veterinary medicine, mycoplasmas represent a major economic burden to the global livestock industry. *Mycoplasma gallisepticum* (*M. gallisepticum*) and *Mycoplasma synoviae* (*M.synoviae*) are considered the most economically significant mycoplasma species in commercial poultry production worldwide, causing chronic respiratory disease, arthritis, and reduced egg production (Feberwee et al., 2025). *Mycoplasma bovis* (*M.bovis*) causes chronic bronchopneumonia, mastitis, arthritis, and reproductive tract disease in cattle globally and is an emerging pathogen in bison, with control being particularly challenging due to limited effective antimicrobial treatments and vaccines (Premachandre et al., 2024). *Mycoplasmasuis* (*M.suis*) is responsible for porcine hemolytic anemia (Hoelzle et al., 2014). The economic impact of these infections extends beyond direct production losses to include costs associated with disease control measures, trade restrictions, and compromised animal welfare (Wagner et al., 2025).

The virulence of mycoplasmas is mediated by diverse factors, including adhesins, invasins, capsules, lipoglycans, membrane lipoproteins, and superantigens (Shimizu, 2015). Adhesion to host cells is a critical first step in pathogenesis, enabling nutrient acquisition and immune evasion; non-adherent strains are typically avirulent (Krause et al., 1982). Many pathogenic mycoplasmas employ a specialized tip organelle, a flask-shaped or filamentous apical structure, to anchor to host cell membranes and trigger cascades of pathogenic events. Notably, *M. pneumoniae* and *M. genitalium* have been extensively studied for their adhesins, which facilitate colonization of respiratory or urogenital epithelia (Chaudhry et al., 2007). Another emerging virulence factor, lipid-associated membrane proteins (LAMPs), exhibits high antigenicity and may mediate host cell adhesion, invasion, and subsequent tissue damage (Bai et al., 2015).

## 2 Mycoplasmas and post-translational modifications

To establish infection and persist within host organisms, microbial pathogens have evolved sophisticated strategies to evade host immune defenses. A key mechanism involves the manipulation of host cell signaling pathways to suppress defensive responses against invading pathogens (Koul et al., 2000). Post-translational modifications (PTMs) represent a crucial regulatory mechanism in this process, enabling the chemical alteration of proteins after translation to modulate their function, stability, and interactions (Nussinov et al., 2012). PTMs significantly expand the functional diversity of bacterial proteomes without requiring extensive genomic expansion, providing an evolutionarily efficient means of adaptation (Prus et al., 2019). Common amino acid targets for PTMs include serine, threonine, aspartate, tyrosine, histidine, asparagine, lysine, and arginine (Hauser et al., 2017), with modifications ranging from small chemical groups (e.g., phosphorylation, acetylation) to complex oligosaccharide structures or polypeptide chain attachments (Greer and Shi, 2012; Macek et al., 2019).

Despite their reduced genomes, mycoplasmas exhibit remarkable adaptability, partly due to PTMs that compensate for their limited transcriptional regulation (Schmidl et al., 2010a). These modifications enhance protein structural complexity and functional precision, enabling mycoplasmas to fine-tune their interactions with host cells (Van Noort et al., 2012). For instance, *M. suis* employs extensive post-translational processing, including cleavage of adhesins, lipoproteins, and moonlighting proteins, to diversify protein functions and expose immunogenic epitopes, thereby increasing the host’s immune burden and facilitating immune evasion (Li et al., 2020).

Given their minimal genomes, mycoplasmas rely on glycolytic fermentation for ATP production, with glucose catabolized to lactate and acetate. Beyond energy generation, the activity of these metabolic pathways is regulated through post-translational mechanisms. Key glycolytic and fermentative enzymes undergo regulatory phosphorylation mediated by protein kinases, enabling *M. pneumoniae* to dynamically adjust metabolic flux in response to environmental changes and compensating for its limited transcriptional regulatory capacity (Atkinson et al., 2008; Yus et al., 2009).

## 3 Major protein PTMs in mycoplasmas

The most prevalent PTMs in mycoplasmas include phosphorylation, acetylation, and glycosylation, which can be either reversible or irreversible (Su et al., 2007; Van Noort et al., 2012; Daubenspeck et al., 2015).

### 3.1 Protein phosphorylation in mycoplasmas

Protein phosphorylation is usually a dynamic and reversible process. Phosphorylation is catalyzed by kinase, while dephosphorylation is catalyzed by phosphatase. They have been described in many pathogenic mycoplasmas as an essential protein involved in phosphorylation-dependent signal transduction pathways and are frequently associated with the virulence of these organisms. Therefore, in the field of protein phosphorylation, the kinase family and their corresponding phosphorylated residues are usually described jointly. Protein phosphorylation, which involves the transfer of a phosphate group from ATP to specific amino acid residues on substrate proteins, is a fundamental post-translational modification (PTM) that regulates diverse cellular processes in both eukaryotes and prokaryotes. In mycoplasmas, phosphorylation is among the most extensively studied PTMs, with significant findings reported in species such as *M. pneumoniae* and *M. genitalium* (Su et al., 2007; Van Noort et al., 2012). This modification primarily targets the hydroxyl groups of serine (Ser), threonine (Thr), and tyrosine (Tyr) residues; however, phosphorylation can also occur on histidine (His), arginine (Arg), lysine (Lys), aspartic acid (Asp), and cysteine (Cys) (**Supplementary Table 1**). Among these, serine and threonine phosphorylations are the most prevalent and well-characterized in mycoplasmas and are often mediated by dual-specificity kinases and phosphatases (Su et al., 2007).

**Figure 1:**
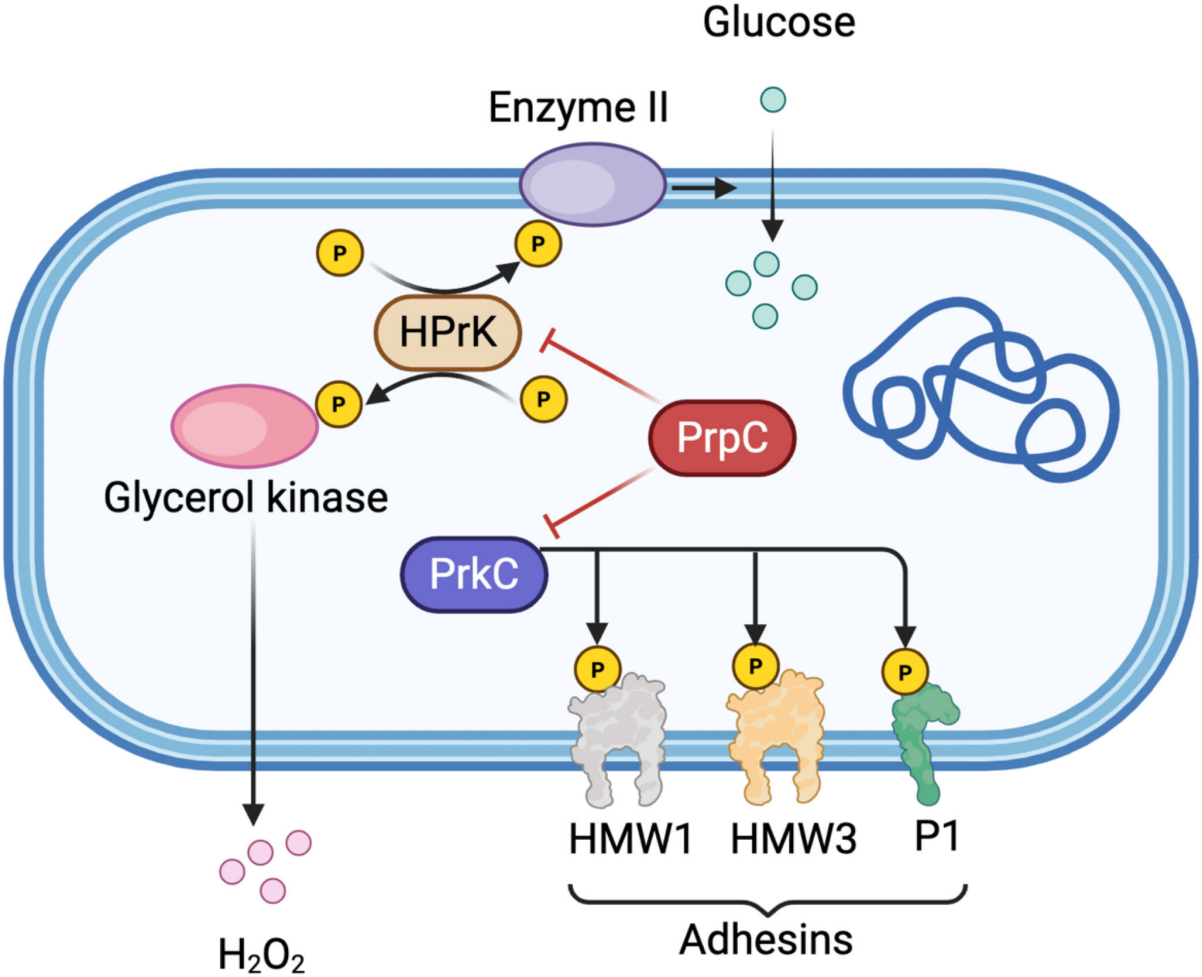
Schematic representation of protein phosphorylation regulatory network in Mycoplasma pneumoniae. The diagram illustrates the key components of phosphorylation-dependent signal transduction in M. pneumoniae. Glucose enters the cell via Enzyme II (EII) of the phosphotransferase system (PTS). HPr kinase (HPrK) undergoes phosphorylation at multiple sites (indicated by yellow circles with “P”) and is activated under low ATP conditions. Glycerol kinase activity is regulated by HPrK-mediated phosphorylation, modulating H_2_O_2_ production as a virulence factor. The serine/threonine kinase PrkC phosphorylates membrane-associated adhesins including HMW1, HMW3, and P1, which are essential for bacterial adhesion and pathogenicity. The phosphatase PrpC (shown in red) counteracts PrkC activity by dephosphorylating its substrates, including HPrK and the adhesin proteins. Red inhibitory arrows indicate negative regulation by PrpC, while black arrows represent phosphorylation events or metabolic flow. This regulatory interplay between kinase and phosphatase activities is critical for maintaining the dynamic balance of protein phosphorylation states and for modulating mycoplasma virulence. Created in BioRender. https://BioRender.com.

Protein phosphorylation is typically a dynamic and reversible process: kinases catalyze the addition of phosphate groups, while phosphatases catalyze their removal. Both kinases and phosphatases have been identified in numerous pathogenic mycoplasmas, where they play critical roles in phosphorylation-dependent signal transduction pathways and are frequently associated with virulence. (**Figure 1**).

#### 3.1.1 Serine/threonine kinases

STKs in bacteria can be broadly classified into eukaryotic-like kinases and atypical bacterial kinases. Although Gram-positive bacteria often encode multiple STKs, mycoplasmas, which are characterized by their highly reduced genomes, typically possess only a limited number. For example, *Mycoplasma pneumoniae* encodes just two kinases: HPr kinase (HPrK) and the serine/threonine-protein kinase PrkC (Schmidl et al., 2010a). Homologs of HPrK have also been identified and functionally detected in *M. capricolum*, *M. genitalium*, and *Acholeplasma laidlawii* (Zhu et al., 1997), underscoring its conservation across mycoplasma species.

In *M. pneumoniae*, HPrK demonstrates enhanced kinase activity under low ATP conditions—a metabolic adaptation likely linked to its niche in the respiratory tract (Allen et al., 2003). Notably, glycerol availability has been shown to trigger HPrK-mediated phosphorylation of glycerol kinase, a regulatory mechanism that may modulate cytotoxic H_2_O_2_ production, a virulence trait notably observed in *M. mycoides* (Halbedel et al., 2004). Phosphorylated HPr subsequently transfers its phosphate group to Enzyme II (EII) components of the phosphotransferase system (PTS), thereby facilitating sugar transport across the membrane (Zhu et al., 1998). These findings suggest that HPrK-dependent phosphorylation serves as a fine-tuning mechanism for substrate utilization, providing insight into how carbon metabolism may influence mycoplasma pathogenicity.

Functional studies indicate that PrkC is essential for the pathogenicity of *M. pneumoniae*. A prkC mutant exhibits significantly impaired adhesion and cytotoxicity, accompanied by altered phosphorylation patterns of key adhesins such as HMW1, HMW3, and P1 (Schmidl et al., 2010a).

#### 3.1.2 Phosphatases

Phosphatases represent a class of reversibly regulated enzymes that play critical roles in diverse cellular processes, such as protein dephosphorylation and translational regulation. Serine/threonine protein phosphatases are classified into three major families based on their primary structure, substrate specificity, and conserved catalytic domains: phosphoprotein phosphatases (PPPs), metal-dependent protein phosphatases (PPMs), and aspartate-based phosphatases, which include FCP/SCP and HAD enzymes. The PPP family is subdivided into seven distinct subfamilies, while the PPM family encompasses magnesium/manganese-dependent phosphatases such as PP2C and pyruvate dehydrogenase phosphatases (Moorhead et al., 2009; Shi, 2009; Brautigan, 2013).

In contrast to kinases, which catalyze protein phosphorylation, phosphatases counter this process through dephosphorylation, thereby ensuring the dynamic reversibility of this key post-translational modification. Recent functional studies have revealed that MG207, one of only three phosphatases in *Mycoplasma genitalium*, modulates the phosphorylation of multiple substrate proteins and is required for full bacterial virulence; its deletion attenuates pathogenicity (Martinez et al., 2013). Furthermore, *Mycoplasma synoviae* carries a gene (*prpC*) predicted to encode a phosphatase of the PP2C subfamily. In *Mycoplasma pneumoniae*, PrpC regulates the phosphorylation state of HPrK, thereby influencing carbon metabolism (Schmidl et al., 2010b). Conversely, inactivation of the *prpC* gene impairs its phosphatase activity, which normally antagonizes PrkC-mediated phosphorylation. This disruption results in enhanced phosphorylation and intracellular accumulation of key adhesion proteins, including HMW1, HMW3, the primary adhesin P1, and the surface protein Mpn474. further underscoring the critical balance between kinase and phosphatase activities in mycoplasma virulence (Schmidl et al., 2010a).

Despite progress, the phosphorylation landscape in mycoplasmas remains incompletely mapped. For example, *M. pneumoniae* proteomics has identified 63 phosphorylated proteins, yet the kinases responsible for most modifications are unknown (Schmidl et al., 2010a). Similarly, *M. gallisepticum* exhibits 15 phosphorylated proteins with uncharacterized regulatory mechanisms (Demina i et al., 2009). Further studies integrating phosphoproteomics and genetic screens will be essential to elucidate the full scope of phosphorylation-dependent virulence mechanisms in these minimal pathogens.

### 3.2 Protein acetylation in mycoplasmas

Protein acetylation has emerged as a critical regulatory mechanism in mycoplasmas, representing a fascinating evolutionary adaptation to their genomic minimalism. While the basic biochemistry of acetylation is conserved across domains of life, mycoplasmas appear to have developed unique dependencies on this modification that warrant special consideration.

The acetylation process involves transfer of an acetyl group from acetyl coenzyme A (Ac-CoA) to target lysine residues, catalyzed by lysine acetyltransferases. Among these enzymes, N-acetyltransferases are particularly significant (Murray, 1964). N-acetyltransferase-mediated reactions proceed through deprotonation of the lysine substrate, enabling nucleophilic attack on the Ac-CoA carbonyl carbon, ultimately transferring the acetyl group to the target residue (Vetting et al., 2005).

#### 3.2.1 Functional roles of acetylation

Protein acetylation in mycoplasmas plays crucial regulatory roles across multiple cellular processes. In metabolic regulation, acetylated proteins are prominently involved in energy metabolism and biosynthetic pathways, with distinct patterns observed between species. Studies have revealed extensive acetylation of key metabolic enzymes in *M. pneumoniae*, including enolase (Eno) and elongation factor Tu (EF-Tu) (Van Noort et al., 2012; Chen et al., 2016), suggesting that acetylation serves as an important mechanism for fine-tuning essential metabolic pathways in these minimal organisms. The modification of central carbon metabolism enzymes coordinates substrate utilization and metabolic flux, providing a streamlined regulatory mechanism to compensate for limited transcriptional control networks.

Beyond metabolism, acetylation significantly impacts genetic information processing in mycoplasmas. Critical DNA-associated proteins such as DNA gyrase (GyrB), RNA polymerase (RpoE), and chromosome segregation protein (ScpA) are frequent acetylation targets. This modification likely influences fundamental processes including transcription initiation, DNA topology maintenance, and chromosomal organization, potentially serving as a versatile regulatory mechanism to compensate for the reduced transcriptional control networks in these genomically minimalist bacteria. The extensive acetylation of core nucleic acid-associated proteins suggests that this PTM may represent a crucial layer of gene expression control in mycoplasmas, enabling rapid functional modulation without the requirement for extensive regulatory protein networks.

**Figure 2:**
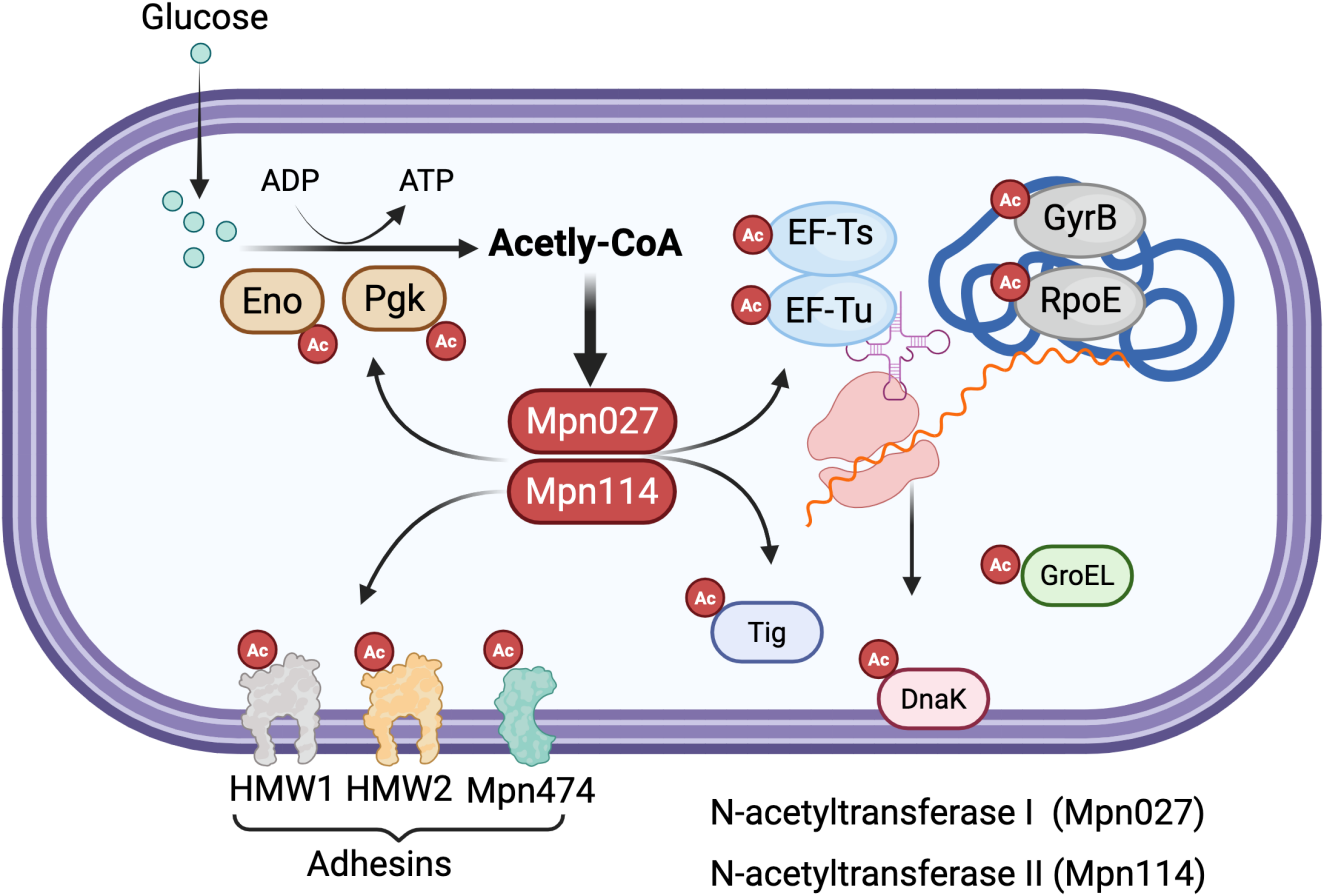
Protein acetylation regulatory network in mycoplasmas. The diagram depicts the acetylation-mediated regulation of diverse cellular processes in mycoplasmas. Glucose metabolism generates acetyl-CoA via glycolysis, with key glycolytic enzymes enolase (Eno) and phosphoglycerate kinase (Pgk) undergoing acetylation (marked by red circles with “Ac”). Two N-acetyltransferases, Mpn027 and Mpn114, catalyze the transfer of acetyl groups from acetyl-CoA to lysine residues on target proteins. Major acetylation targets include (Dallo and Baseman, 2000): metabolic enzymes (Eno, Pgk) involved in energy production; (2) translation machinery components (elongation factors EF-Ts and EF-Tu); (3) genetic information processing proteins including DNA gyrase subunit B (GyrB) and RNA polymerase subunit (RpoE); (4) protein homeostasis factors such as the trigger factor chaperone (Tig), GroEL chaperonin, and DnaK chaperone; and (5) membrane-associated adhesins (HMW1, HMW2, and Mpn474) critical for host cell attachment. This widespread acetylation pattern across metabolic, genetic, and virulence-related pathways highlights the central regulatory role of protein acetylation in coordinating cellular processes in genomically minimal mycoplasmas. Created in BioRender. https://BioRender.com.

Protein homeostasis represents another key functional domain affected by acetylation in mycoplasmas. Molecular chaperones including the trigger factor (Tig) and various protein turnover components show preferential acetylation (Van Noort et al., 2012; Chen et al., 2016), indicating that this modification may modulate protein folding capacity, stability, and degradation pathways. The modification of chaperones such as GroEL/GroES and DnaK systems suggests that acetylation can influence protein quality control mechanisms, potentially affecting the functional states of client proteins. These findings collectively establish acetylation as a multifaceted regulatory system in mycoplasmas, coordinating diverse cellular processes from metabolism to genetic information flow and protein quality control through a relatively simple yet effective post-translational modification mechanism that may partially compensate for their limited regulatory gene repertoire (**Figure 2**).

#### 3.2.2 Regulatory networks

Recent proteomic studies have uncovered an unexpectedly extensive landscape of protein acetylation in mycoplasmas, revealing the remarkable scope of this modification system. Comprehensive analyses have identified 3,045 acetylated lysine residues in *M. pneumoniae* and 4,156 in *M. genitalium* (**Supplementary Table 2**), representing a substantial fraction of the lysine residues in these proteomes. Particularly noteworthy is the observation that acetylation sites significantly outnumber phosphorylation sites in both species (Chen et al., 2016), suggesting that lysine acetylation may serve as a predominant post-translational modification system in these genomically reduced organisms. This extensive acetylation network potentially compensates for the limited repertoire of regulatory proteins through this versatile chemical modification strategy, enabling sophisticated control of protein function despite severe genomic constraints. The prevalence of acetylation in mycoplasmas underscores its fundamental importance in maintaining cellular homeostasis and adapting to environmental changes in organisms with minimal genetic complexity.

Emerging evidence reveals a sophisticated interplay between different post-translational modifications in mycoplasmas, where phosphorylation networks appear to influence acetylation states while evolutionarily conserved proteins and metabolic enzymes demonstrate particular susceptibility to lysine acetylation (Van Noort et al., 2012). These findings establish acetylation as a pivotal regulatory mechanism governing mycoplasma physiology, with the extensive modification of metabolic enzymes suggesting its crucial role in maintaining energy homeostasis within these genomically streamlined pathogens. The strategic targeting of these acetylation-dependent regulatory networks may offer promising therapeutic avenues for combating mycoplasma infections, particularly given their potential influence on virulence and host adaptation mechanisms, warranting further investigation into their precise roles in pathogenesis.

#### 3.2.3 Unexplored aspects of mycoplasma acetylation

The field of mycoplasma acetylation research presents several critical unanswered questions that warrant further investigation. Key knowledge gaps include understanding the temporal dynamics of acetylation during different infection stages, determining whether mycoplasma acetylation enzymes exhibit unique substrate specificities compared to other bacteria, elucidating potential connections between acetylation and virulence, and exploring how mycoplasma acetylation might influence host immune responses. These unresolved questions highlight the need for systematic studies of acetylation dynamics during infection, development of specialized genetic tools to investigate specific acetylation sites, evaluation of acetylation inhibitors as potential therapeutics, and comparative analyses across mycoplasma species to identify conserved regulatory patterns.

The widespread acetylation observed in mycoplasmas likely represents a fundamental adaptive strategy that allows these genomically reduced organisms to maintain sophisticated regulatory control despite their minimal genetic repertoire. This modification system may serve as an evolutionary solution to compensate for the loss of more complex regulatory networks found in other bacteria. Deciphering the mechanisms and functions of mycoplasma acetylation could yield transformative insights into bacterial adaptation strategies while simultaneously revealing novel targets for therapeutic intervention against these persistent pathogens. The potential dual significance of these findings, for both understanding bacterial evolution and developing new antimicrobial approaches, makes this an exceptionally promising area for future research.

### 3.3 Protein glycosylation in mycoplasmas

Protein glycosylation constitutes a ubiquitous post-translational modification that dramatically expands proteomic diversity across all domains of life (Nothaft and Szymanski, 2010; Nothaft and Szymanski, 2013). This evolutionarily ancient process orchestrates numerous critical cellular functions, including protein folding and stability (Helenius and Aebi, 2004), host-pathogen recognition, and virulence modulation (Valguarnera et al., 2016). While eukaryotic glycosylation predominantly governs protein quality control and intercellular signaling, bacterial glycosylation systems have emerged as key determinants of pathogenicity (Guerry et al., 2006).

Bacterial protein glycosylation operates through three principal mechanisms: N-linked glycosylation, characterized by glycan attachment to asparagine or arginine residues; O-linked glycosylation, involving sugar conjugation to serine/threonine hydroxyl groups; and S-linked glycosylation, mediated through cysteine thiol modifications (Young et al., 2002). In most bacterial systems, UDP-N-acetylglucosamine functions as the predominant glycosyl donor (Bernatchez et al., 2005), with glycosyltransferases, which include recently identified prokaryotic-specific variants, catalyzing these modifications through direct or indirect biosynthetic pathways.

#### 3.3.1 Unconventional glycosylation architecture in genomically minimal mycoplasmas

Mollicute metabolism centers on two fundamental survival strategies: nutrient parasitism of host cells and sophisticated immune evasion. As bacterial symbionts lacking a cell wall, Mollicutes establish persistent infections through multifaceted immune avoidance mechanisms, including dynamic surface proteomes featuring phase-variable proteins and extensive proteolytic processing (Daubenspeck et al., 2015). Despite their drastically reduced genomes encoding only sparse glycosyltransferase and nucleotidyltransferase repertoires (Jordan et al., 2013), mycoplasmas have retained surprisingly sophisticated glycosylation capabilities that generate structurally diverse polysaccharides, immunomodulatory glycolipids, and heterogeneous glycoproteins. GT2 family glycosyltransferases, encoded by essential genes in pathogenic species, constitute the core glycosylation machinery (Andres et al., 2011). Comprehensive proteomic analyses have confirmed widespread O-linked glycosylation at serine and threonine residues across multiple mycoplasma species (**Supplementary Table 3**) (Daubenspeck et al., 2015; Sanford et al., 2025). The evolutionary conservation of these systems in organisms operating under extreme genetic constraints underscores that glycosylation fulfills indispensable biological functions that remain non-redundant even in minimalist genomes (Sanford et al., 2025).

#### 3.3.2 Paradigm-shifting discoveries: a unique hexosylation system

Groundbreaking work in murine pathobionts *M. arthritidis* and *M. pulmonis* has revealed a surface protein glycosylation system fundamentally distinct from all previously characterized bacterial glycosylation pathways. This unconventional hexosylation system attaches hexoses through both N-linkages (to asparagine and glutamine residues) and O-linkages (to serine and threonine residues). Remarkably, isotope labeling experiments demonstrated that hexoses are cleaved directly from exogenous oligosaccharides rather than being transferred from nucleotide sugar donors as in canonical Leloir glycosyltransferase pathways (Jordan et al., 2013; Szymanski, 2022). This metabolically elegant mechanism harnesses the energy stored within glycosidic bonds to drive the glycosylation reaction, catalyzed by as-yet-unidentified enzyme(s).

A defining characteristic of this system is its extraordinary promiscuity: hexosylation occurs without discernible amino acid consensus sequences flanking modification sites, resulting in extensive glycoprotein heterogeneity across the mycoplasmal cell surface (Daubenspeck et al., 2015). Recent high-resolution mass spectrometry studies have expanded this paradigm further, revealing that *M. pulmonis* and *M. arthritidis* can also glycosylate tyrosine residues and, most surprisingly, the acidic amino acids aspartic acid and glutamic acid, modifications not previously described in any bacterial system. The system demonstrates remarkable substrate versatility, utilizing disaccharides with both α- and β-linkages and attaching predominantly glucose with minor amounts of mannose scavenged from host-derived oligosaccharides (Sanford et al., 2025).

The identification of this hexosylation system in *M. genitalium* (580-kbp genome), the ruminant pathogen *M. mycoides* subsp. *capri*, and the synthetic minimal organism JCVI-Syn3A (543-kbp genome) provides compelling evidence for its essentiality. Syn3A’s rationally designed genome retains only genes critical for robust growth under axenic conditions, yet hexosylation machinery was preserved, strongly validating the system’s fundamental importance even in laboratory settings (Sanford et al., 2025).

Additionally, *M. genitalium* possesses MG517, a multifunctional glycosyltransferase capable of modifying both nascent and pre-glycosylated lipid substrates (Romero-garcia et al., 2019). These discoveries not only overturn conventional paradigms regarding glycosylation capacity limitations in genome-reduced organisms but also illuminate the profound evolutionary importance of these modifications in mycoplasma biology.

#### 3.3.3 Functional significance and protective roles of hexosylation

The retention of hexosylation systems in genomically streamlined mycoplasmas illuminates fundamental principles of metabolic adaptation to parasitic lifestyles. Mycoplasmas cannot synthesize most amino acids *de novo* due to extensive genome reduction and must instead scavenge host proteins for essential amino acids. Consequently, these organisms produce an abundance of secreted proteases presumed essential for nutrient acquisition and host survival (Ganter et al., 2019; Gaurivaud and Tardy, 2022). However, this proteolytic strategy poses a potential threat of self-digestion.

Post-translational modifications, including glycosylation, can sterically hinder protease accessibility in the immediate vicinity of modification sites (Alemka et al., 2013; Sanford et al., 2025). We therefore propose that hexosylation serves a critical protective function: shielding mycoplasmal surface proteins from degradation by endogenous proteases (Daubenspeck et al., 2015). This hypothesis is supported by the observation that glycosylated peptides resist tryptic cleavage at nearby lysine residues, and most identified glycosites cluster near potential protease recognition sites. The apparent stochasticity of glycosylation, with individual protein molecules modified at different sites, would provide probabilistic protection across the proteome while maintaining functional protein diversity.

**Figure 3:**
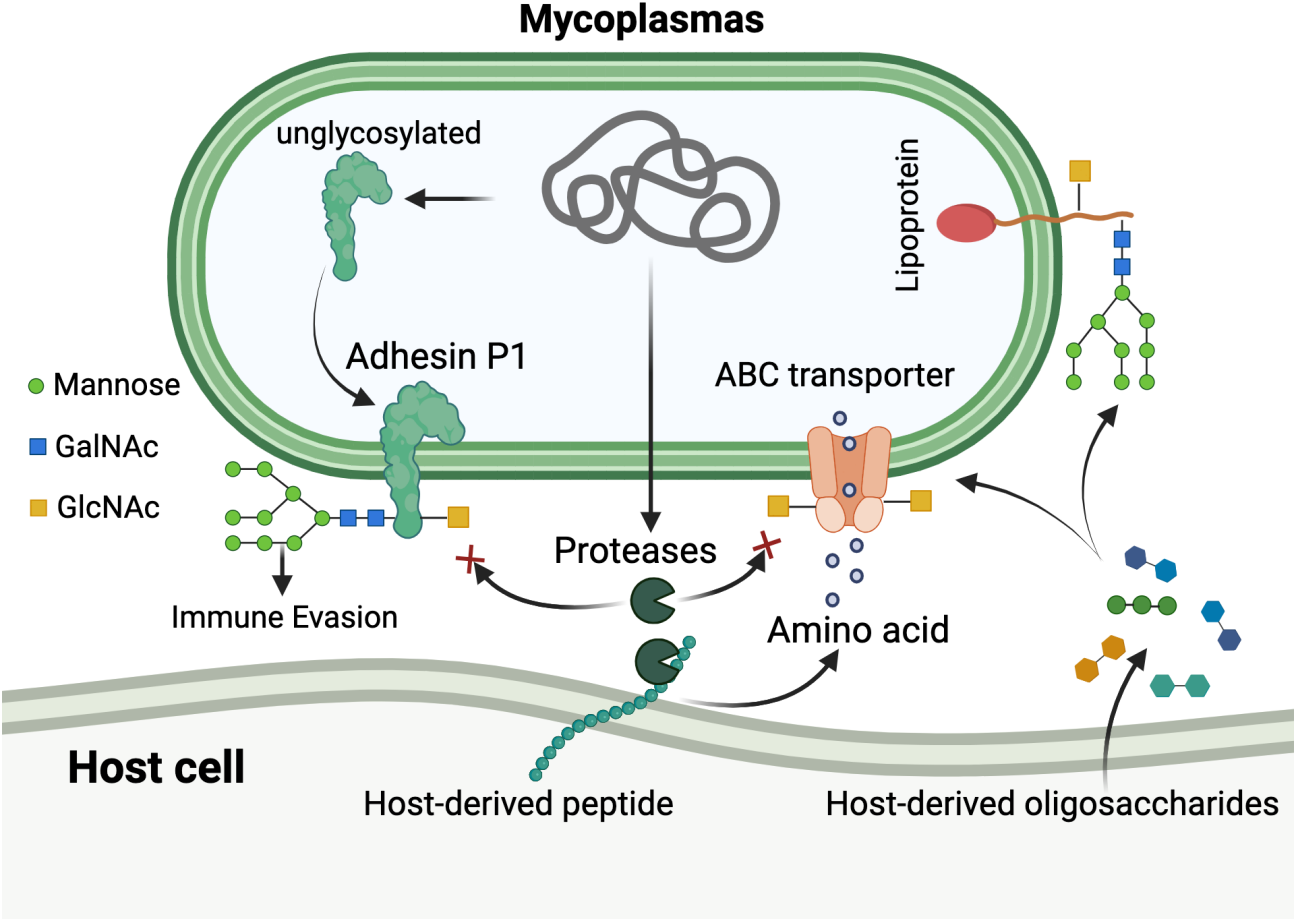
Unconventional hexosylation system and its functional roles in mycoplasmas. The diagram illustrates the unique glycosylation mechanism in mycoplasmas. Host-derived oligosaccharides and peptides are transported into the mycoplasmal cell via ABC transporters and endogenous proteases. Through a non-canonical hexosylation pathway, mycoplasmas cleave hexoses (primarily glucose, shown as green circles; and mannose) directly from host-derived oligosaccharides without utilizing nucleotide sugar donors. These hexoses are then attached to diverse amino acid residues on mycoplasmal proteins through both N-linkages (asparagine, glutamine) and O-linkages (serine, threonine, tyrosine, aspartic acid, and glutamic acid), generating extensive glycoprotein heterogeneity. Glycosylation patterns are shown with various sugar compositions including mannose (green circles), N-acetylgalactosamine (GalNAc, blue squares), and N-acetylglucosamine (GlcNAc, yellow squares). The modification occurs without consensus sequence requirements, resulting in stochastic glycosylation across surface proteins including the major adhesin P1. This system provides multiple adaptive advantages: (1) protection of surface proteins from degradation by endogenous proteases (indicated by red inhibition symbols); (2) immune evasion through surface antigenic variation; and (3) molecular mimicry of host glycosylation patterns for immunological camouflage. The lipoprotein shown on the right represents membrane-anchored glycoproteins with diverse glycan structures derived from host sources. Created in BioRender. https://BioRender.com.

Beyond self-protection, we propose three additional synergistic selective advantages (Dallo and Baseman, 2000): functional economy, whereby multifunctional glycan modifications substitute for dedicated protein effector systems (Principi and Esposito, 2001); adaptive surface plasticity, compensating for limited membrane proteome diversity through variable glycosylation patterns that generate extensive antigenic heterogeneity, potentially representing a novel form of immune evasion contributing to chronic infection; and (Dybvig and Voelker, 1996) immunological camouflage, enabling surface mimicry of host glycosylation patterns through utilization of host-derived oligosaccharides, potentially reducing immunogenicity (**Figure 3**).

#### 3.3.4 Outstanding questions and therapeutic implications

While these studies were conducted under axenic culture conditions, hexosylation likely modulates diverse host-mycoplasma interactions during infection, including immune recognition, cellular adhesion, and biofilm formation. Critical knowledge gaps persist regarding (1): the molecular identity and catalytic mechanism of the glycosyltransferase(s) responsible for this unconventional hexosylation (2); structural determinants governing substrate recognition without consensus sequences (3); quantitative dynamics of glycosylation heterogeneity within mycoplasma populations; (4) precise functional contributions during host colonization and immune evasion; and (5) potential roles of horizontal gene transfer in maintaining these systems across species.

### 3.4 Other post-translational modifications in mycoplasma

#### 3.4.1 Lipid modifications: palmitoylation

Among the various PTMs identified in mycoplasmas, lipid modifications represent an important regulatory mechanism. Palmitoylation, the covalent attachment of palmitic acid to proteins, has been documented in several Mollicutes species. In *Ureaplasma urealyticum* serotype 8, approximately 25 palmitoylated proteins were detected through metabolic labeling with [³H]palmitic acid, with at least six of these being antigenic proteins, including the serotype 8-specific 96-kDa surface antigen (Thirkell et al., 1991). Phase partitioning experiments demonstrated that the majority of these acylated proteins were membrane-associated, with some displaying amphipathic properties (Thirkell et al., 1991).

Similar lipid modifications have been reported in other mycoplasma species. In *Mycoplasma hyopneumoniae*, major membrane surface proteins were found to be selectively modified by covalently bound lipids (Wise and Kim, 1987). *Mycoplasma hyorhinis* exhibits lipid-modified surface antigens that express size variation, potentially serving as a mechanism for generating antigenic diversity and mediating immune responses (Boyer and Wise, 1989). In *Mycoplasma capricolum*, proteolipid formation was influenced by cholesterol availability and unsaturated fatty acid acylation patterns (Dahl et al., 1983). Notably, in *Acholeplasma laidlawii*, selective acylation of membrane proteins has been observed, with palmitoylation typically occurring through thioester linkages to cysteine residues (Nyström et al., 1986).

The functional significance of protein palmitoylation in mycoplasmas likely extends beyond simple membrane anchoring. It has been proposed that lipid modification may play crucial roles in dictating host-pathogen interactions, affecting organism pathogenicity, and modulating immune responses that influence growth and survival (Boyer and Wise, 1989; Thirkell et al., 1991). The identification of palmitoylated surface antigens in *U. urealyticum* suggests that this modification may be involved in host cell recognition and adhesion processes critical for colonization.

#### 3.4.2 Methylation: evidence and gaps

While protein methylation has been extensively documented as a regulatory PTM in both eukaryotes and prokaryotes, direct evidence for protein methylation in mycoplasmas remains limited. The most comprehensive data available concerns DNA methylation rather than protein methylation. Single-molecule real-time (SMRT) sequencing has revealed complete methylomes for *M. genitalium* and *M. pneumoniae* at single-base resolution, identifying novel N^6^-methyladenine (6mA) methylation motifs associated with restriction-modification systems (Lluch-Senar et al., 2013). These studies detected multiple DNA methyltransferases, including MPN198 and MPN343 in *M. pneumoniae*, which are responsible for specific methylation patterns (Lluch-Senar et al., 2013).

However, evidence for protein methylation as a PTM in mycoplasmas is largely indirect. Large-scale proteomic studies in *M. pneumoniae* have demonstrated extensive cross-talk between phosphorylation and lysine acetylation (Van Noort et al., 2012), but protein methylation was not specifically investigated in these studies. Theoretical considerations suggest that protein methylation should occur in mycoplasmas, as the enzymes and substrates are present. In bacterial chemotaxis systems, methylation and phosphorylation have been shown to collectively regulate signaling pathways (Wuichet and Zhulin, 2010), and similar mechanisms may operate in mycoplasmas. Additionally, in the genome-reduced bacterium *M. pneumoniae*, several putative methyltransferases are encoded in the genome and are regulated by protein degradation machinery (Burgos et al., 2020), suggesting potential roles in protein modification beyond DNA methylation.

The absence of direct experimental evidence for protein methylation in mycoplasmas may reflect technical challenges rather than biological absence. The limited number of comprehensive PTM studies in these organisms, combined with the technical difficulties of metabolic labeling (as evidenced by the challenges encountered in palmitoylation studies (Thirkell et al., 1991)), may have prevented detection of this modification. Future studies employing sensitive mass spectrometry-based approaches for PTM identification would be valuable for definitively establishing whether protein methylation occurs in mycoplasmas.

#### 3.4.3 SUMOylation: an unlikely modification in mycoplasmas

SUMOylation (Small Ubiquitin-like Modifier modification) is a well-characterized PTM in eukaryotes that regulates protein function, localization, and stability. However, there is currently no evidence for SUMOylation in mycoplasmas or prokaryotes more broadly. The SUMO conjugation system requires a specific enzymatic machinery, including E1 activating enzymes, E2 conjugating enzymes, and E3 ligases, which are characteristic features of eukaryotic cells (Zhao, 2018).

Genome analysis of sequenced mycoplasma species, including the well-characterized *M. pneumoniae* genome (approximately 816 kb encoding 688 proteins), reveals no homologs of SUMO or the associated conjugation machinery (Dandekar et al., 2000). The minimal genomes of mycoplasmas, which have undergone extensive reductive evolution and lost many metabolic pathways, make the presence of such a complex regulatory system highly unlikely.

It should be noted that while classical SUMOylation appears absent, prokaryotes possess functionally analogous systems. Bacterial ubiquitin-like protein (Pup) modification has been identified in actinobacteria, serving similar regulatory functions (Pearce et al., 2008). However, no evidence for Pup or related systems has been reported in mycoplasmas. The evolutionary loss of such modification systems in mycoplasmas is consistent with their strategy of parasitic/commensal lifestyle and reliance on host-derived metabolites.

#### 3.4.4 Conclusions and future directions

Current evidence indicates that mycoplasmas employ a selective set of PTMs, with palmitoylation clearly documented and phosphorylation and acetylation extensively characterized in *M. pneumoniae* (Van Noort et al., 2012). The existence of protein methylation in mycoplasmas remains an open question requiring direct experimental investigation. SUMOylation appears biologically implausible given the absence of required enzymatic machinery and the organisms’ reduced genomic capacity.

Future research should employ comprehensive mass spectrometry-based proteomics approaches to systematically catalog PTMs in mycoplasmas. Such studies would benefit from enrichment strategies specific for methylated peptides and other modifications. Understanding the full repertoire of PTMs in these minimal organisms will provide insights into fundamental regulatory mechanisms and may reveal novel therapeutic targets for mycoplasma-associated diseases.

## 4 PTM resources, tools, and prediction methods in mycoplasma studies

### 4.1 Overview of PTM databases and resources

The comprehensive characterization of post-translational modifications in *Mycoplasma* sp*ecies* requires access to diverse bioinformatics resources and analytical tools. Several major databases serve as repositories for PTM information, each with distinct strengths and coverage characteristics.

#### 4.1.1 General PTM databases

PhosphoSitePlus represents one of the most comprehensive resources for phosphorylation data, containing over 230,000 phosphorylation sites with kinase-substrate relationship annotations (Hornbeck et al., 2015). This database has been successfully utilized in Mycoplasma studies, though it shows variable coverage across different organisms (Huckstep et al., 2021). UniProt serves as a fundamental resource, providing curated PTM annotations including phosphorylation sites marked with the keyword “Phosphoprotein” (KW-0597), which has been extensively used for validating experimental findings in prokaryotic systems (Bateman et al., 2017).

qPhos is a quantitative phosphoproteomics database that contains nearly 200,000 non-redundant phosphorylation sites with cell-type and temporal information from 191 publications across various experimental conditions (Yu et al., 2019). While primarily focused on eukaryotic systems, it provides valuable reference data for comparative analyses. Other phosphorylation-focused databases include PHOSIDA (Gnad et al., 2007), Phospho.ELM (Diella et al., 2004), and RegPhos (Huang et al., 2014), which offer complementary information on phosphorylation sites and kinase predictions.

#### 4.1.2 Pathway and signaling databases

For functional interpretation of PTM data, pathway-oriented databases are essential. Reactome provides highly curated pathway information with specific attention to protein phosphorylation states, treating differently phosphorylated protein forms as distinct entities with unique functions (Joshi-Tope et al., 2005). The Kyoto Encyclopedia of Genes and Genomes (KEGG) offers broad pathway coverage but with limited phosphosite-specific annotations (Kanehisa and Goto, 2000). SIGNOR (SIGnaling Network Open Resource) contains detailed information on causal relationships between biological entities, including 4,923 human phosphorylation annotations with high sequence consistency (98.5%) (Perfetto et al., 2016).

WikiPathways provides community-curated pathway information with growing coverage of PTM-related data, though phosphosite extraction remains challenging due to variable annotation formats (Slenter et al., 2018). For protein-protein interaction data, BioGRID (Stark et al., 2006) and the Human Protein Reference Database (HPRD) (Keshava Prasad et al., 2009) offer extensive networks, with HPRD showing particularly high coverage of phosphorylation events (31,389 phosphorylations with 98.4% sequence consistency) (Huckstep et al., 2021).

#### 4.1.3 Specialized prokaryotic PTM resources

The integration of multi-omics databases specifically designed for bacterial systems has proven valuable for Mycoplasma studies. The MyMpn database represents a comprehensive resource specifically for *Mycoplasma pneumoniae*, integrating 1,748 datasets including DNA methylomes, transcriptomes, proteomes, protein-protein interaction networks, PTMs, metabolomes, and genome-wide essentiality maps (Wodke et al., 2015). This resource has enabled systematic analysis of how acetylation, phosphorylation, and other PTMs contribute to proteome regulation in genome-reduced bacteria (Chen et al., 2016). MyMpn data is stored within a relational database management system, MySQL (https://www.mysql.com).

### 4.2 Analytical tools and workflows

#### 4.2.1 Mass spectrometry-based identification

Modern phosphoproteomics workflows rely heavily on high-resolution mass spectrometry combined with PTM enrichment strategies. Anti-acetyllysine antibodies and anti-phosphotyrosine/phosphoserine/phosphothreonine antibodies enable selective enrichment of modified peptides prior to LC-MS/MS analysis (Christensen et al., 2019). Several quantification approaches are available:

##### 4.2.1.1 Stable isotope labeling

SILAC enables precise quantification through metabolic labeling, though its application in prokaryotes requires careful optimization (Weinert et al., 2013).

##### 4.2.1.2 Chemical labeling

TMT (Tandem Mass Tags) and iTRAQ allow multiplexed quantitative analysis across multiple conditions, particularly useful for time-course experiments (Weinert et al., 2013).

##### 4.2.1.3 Label-free quantification

MS1 filtering combined with data-dependent or data-independent acquisition (DIA) provides cost-effective quantification without isotopic labeling requirements [88.89].

#### 4.2.2 Stoichiometry determination

A critical advancement in PTM analysis has been the development of methods to determine modification stoichiometry—the fraction of a protein population bearing a specific modification. This information is essential for distinguishing functionally relevant modifications from low-occupancy events. Several workflows have been developed that compare endogenous “light” modifications to stable isotope-labeled “heavy” modifications introduced through *in vitro* peracetylation or perphosphorylation of unmodified residues (Baeza et al., 2014; Nakayasu et al., 2014; Meyer et al., 2016).

#### 4.2.3 Bioinformatics analysis platforms

For data analysis and visualization, several software tools are available:

##### 4.2.3.1 Skyline

Enables platform-independent, label-free quantitation of proteomic data using MS1 extracted ion chromatograms, particularly useful for PTM analysis (Schilling et al., 2012).

##### 4.2.3.2 MaxQuant

Provides comprehensive analysis of mass spectrometry data with built-in algorithms for PTM site localization and quantification (Weinert et al., 2013).

##### 4.2.3.3 ClusterProfiler and ROAST

R packages for gene set enrichment analysis and pathway analysis, commonly used for functional interpretation of PTM datasets (Wu et al., 2010; Yu et al., 2012).

#### 4.2.4 Consistency validation tools

Given the challenges of PTM annotation accuracy, tools for validating modification sites against reference sequences are essential. Automated approaches like ProtMapper have been developed to normalize phosphosite information and resolve inconsistencies between databases and protein sequences, addressing issues such as isoform-specific modifications and sequence offsets (Bachman et al., 2019).

### 4.3 Prediction methods for PTM sites

#### 4.3.1 Kinase-substrate prediction

Several computational approaches predict kinase-substrate relationships based on sequence motifs, structural information, and evolutionary conservation:

##### 4.3.1.1 GPS (group-based prediction system)

Predicts kinase-specific phosphorylation sites using group-based scoring algorithms (Huang et al., 2014).

##### 4.3.1.2 NetPhos/NetPhosK

Neural network-based predictors for phosphorylation sites with kinase-family specificity (Gnad et al., 2007).

##### 4.3.1.3 PhosphoNetworks

Integrates experimental data with computational predictions to construct kinase-substrate networks (Huang et al., 2014).

#### 4.3.2 Acetylation site prediction

For lysine acetylation, prediction tools are less developed for prokaryotes compared to eukaryotes, but several approaches show promise (1):Sequence-based prediction using support vector machines (SVM) trained on experimentally validated acetylation sites (2). Structure-based approaches that consider the local protein environment and accessibility of lysine residues (Christensen et al., 2019).

#### 4.3.3 Functional impact prediction

Beyond site prediction, several methods assess the potential functional impact of PTMs:

##### 4.3.3.1 Evolutionary conservation analysis

Sites conserved across species are more likely to be functionally important, though conservation rates vary (35-65% for phosphosites) (Needham et al., 2019).

##### 4.3.3.2 Structural context analysis

Position relative to active sites, binding interfaces, or regulatory domains can indicate functional relevance (Schilling et al., 2015).

##### 4.3.3.3 Network-based approaches

Integration with protein-protein interaction networks and pathway databases enables prediction of PTM effects on cellular processes (Sacco et al., 2018).

### 4.4 Integration strategies and best practices

#### 4.4.1 Multi-database integration

Comparative analysis of seven major databases (Reactome, KEGG, WikiPathways, SIGNOR, HPRD, BioGRID, and PhosphoSitePlus) reveals that no single resource provides comprehensive coverage (Huckstep et al., 2021). Different databases excel at different levels:

##### 4.4.1.1 Protein-level coverage

BioGRID shows highest coverage (>18,000 proteins), followed by Reactome and HPRD (Huckstep et al., 2021).

##### 4.4.1.2 Phosphosite-level coverage

HPRD maintains the most phosphorylation annotations (31,389), with high sequence consistency (98.4%) (Huckstep et al., 2021).

##### 4.4.1.3 Functional annotation

Reactome provides the richest mechanistic information, treating different phosphorylation states as distinct entities (Joshi-Tope et al., 2005).

The limited overlap between databases (only 5% of proteins common to all seven) emphasizes the importance of consulting multiple resources (Huckstep et al., 2021). For Mycoplasma studies, integrating specialized resources like MyMpn with general databases provides optimal coverage (Wodke et al., 2015; Chen et al., 2016).

#### 4.4.2 Analytical workflow recommendations

Based on comparative analyses, an optimal workflow for Mycoplasma PTM analysis should include:

##### 4.4.2.1 Initial protein mapping

Use databases with high proteome coverage (BioGRID, Reactome) to maximize mapping success (Huckstep et al., 2021).

##### 4.4.2.2 Phosphosite annotation

Cross-reference with HPRD, PhosphoSitePlus, and SIGNOR for comprehensive phosphorylation information (Hornbeck et al., 2015; Perfetto et al., 2016; Huckstep et al., 2021).

##### 4.4.2.3 Sequence validation

Verify all PTM sites against current UniProt sequences, allowing for small offsets (± 2 residues) to account for annotation inconsistencies (Bateman et al., 2017; Bachman et al., 2019).

##### 4.4.2.4 Functional interpretation

Integrate pathway information from Reactome and SIGNOR to understand modification consequences on cellular processes (Joshi-Tope et al., 2005; Perfetto et al., 2016).

##### 4.4.2.5 Kinase prediction

Utilize kinase-substrate networks from PhosphoSitePlus and RegPhos to infer regulatory mechanisms (Huang et al., 2014; Hornbeck et al., 2015).

## 5 Summary and future perspectives

The complex interplay of multiple PTMs in mycoplasmas presents both challenges and opportunities for understanding their functional significance. While current proteomic approaches can map modification sites, establishing direct structure-function relationships requires innovative methodologies combining quantitative mass spectrometry with targeted protein analysis and functional validation. Future efforts should focus on developing more sensitive detection techniques and comprehensive PTM databases to systematically characterize these modifications across different mycoplasma species.

Deciphering the biological roles of PTMs in mycoplasmas demands a multi-faceted approach examining their effects on protein stability, activity, and interaction networks. Particularly crucial is understanding how PTM crosstalk regulates key virulence factors and metabolic pathways in these minimal organisms. Such investigations could reveal novel therapeutic targets by identifying essential modification events or enzymes that could be selectively inhibited to disrupt mycoplasma pathogenesis.

The study of mycoplasma PTMs offers unique insights into evolutionary adaptations of genomically reduced pathogens, while providing model systems for understanding post-translational regulation under extreme genetic constraints. As research progresses, integrating structural biology, systems proteomics, and genetic manipulation will be essential for translating PTM discoveries into practical applications, from improved diagnostics to innovative treatment strategies against these persistent pathogens. These advances may ultimately yield broader principles applicable to other microbial systems and cellular processes.
