# Supplementary material for: Survival strategies of mycoplasmas: the critical role of post-translational modifications
Source: Front Cell Infect Microbiol. 2025 Nov 26;15:1688880. doi: 10.3389/fcimb.2025.1688880 (PMC12689956; doi:10.3389/fcimb.2025.1688880)
Supplement: Supplementary file 1 [file Table1.docx]

| **Supplementary Table 2. Identified Acetylation Sites in Mycoplasmas** | | | |
| --- | --- | --- | --- |
| Uniprot ID | Systematic name | Protein name | Acetylated Residue (s) |
| P09924 | Mpn001 | DnaN | K101, K129, K135, K2, K229, K6 |
| P0CJ81 | Mpn003 | GyrB | K308, K579 |
| P0CJ81 | Mpn004 | GyrA | K286, K736 |
| P11311 | Mpn005 | SerS | K36, K51, K78, K415 |
| P22446 | Mpn015 | Mpn015 | K22 |
| P22446 | Mpn018 | Mpn018 | K576 |
| P22446 | Mpn020 | Mpn020 | K252, K260, K350, K765, K793, K923 |
| P22447 | Mpn021 | DnaJ | K24, K378, K385 |
| P23568 | Mpn025 | Fba | K24, K54, K165, K248, K256 |
| P23568 | Mpn027 | Mpn027 | K256 |
| P41205 | Mpn029 | Efp | K8, K152 |
| P41205 | Mpn030 | Mpn030 | K106 |
| P46775 | Mpn034 | PolC | K197, K1411 |
| P53527 | Mpn044 | Tdk | S2 |
| P54125 | Mpn045 | HisS | K71 |
| P54125 | Mpn046 | AspS | K281 |
| P54125 | Mpn050 | GlpK | K341 |
| P54125 | Mpn051 | Mpn051 | K64, K130, K290, K298, K368 |
| P57079 | Mpn053 | PtsH | K30, K65, K84 |
| P75033 | Mpn055 | PotA | K190, K259, K261, K265 |
| P75039 | Mpn059 | Gcp | K207 |
| P75039 | Mpn061 | Ffh | K175, K276, K311, K413 |
| P75040 | Mpn062 | DeoD | K170, K416, K420 |
| P75041 | Mpn063 | DeoC | K2, K35, K179 |
| P75042 | Mpn064 | ManB | M1 |
| P75043 | Mpn066 | Mpn066 | K535 |
| P75044 | Mpn067 | Mpn067 | K84, K306, K519, K641 |
| P75045 | Mpn078 | FruA | K133, K259 |
| P75046 | Mpn082 | Tkt | K14, K37, K82, K114 |
| P75048 | Mpn105 | PheS | K24, K93 |
| P75049 | Mpn109 | Mpn109 | K21, K24, K34, K43, K197, K666, K900 |
| P75050 | Mpn115 | InfC | K32, K64, K96, K281, K350, K458, K492 |
| P75051 | Mpn119 | GrpE | K3, K355, K549 |
| P75052 | Mpn120 | Mpn120 | K88, K118, K211, K225, K721, K732 |
| P75053 | Mpn121 | Mpn121 | K13, K43, K162, K379, K383, K387, K574 |
| P75054 | Mpn123 | ParC | K32, K50, K65, K79, K204, K280, K384, K416, K428 |
| P75055 | Mpn134 | Mpn134 | K13, K27, K73, K135 |
| P75059 | Mpn153 | Mpn153 | K8, K46, K103, K116, K165, K233 |
| P75061 | Mpn154 | NusA | K24, K266, K28, K360, K63, K101, K147 |
| P75063 | Mpn155 | InfB | K28, K64, K89, K109, K122, K237, K316, K507 |
| P75064 | Mpn157 | Mpn157 | K748, K879, K891, K914 |
| P75067 | Mpn161 | Mpn161 | K132, K636, K740 |
| P75068 | Mpn162 | RpsJ | K101, K268, K272, K275, K305, K31 |
| P75069 | Mpn164 | RplC | K6, K233, K268 |
| P75070 | Mpn165 | RplD | K124, K136, K139, K462, K496 |
| P75079 | Mpn166 | RplW | K197, K587 |
| P75080 | Mpn167 | RplC | K24, K19, K107, K202, K4, K426, K687 |
| P75081 | Mpn169 | RpsS | K112, K98 |
| P75083 | Mpn170 | RplD | K686 |
| P75084 | Mpn171 | RpsC | K367, K604, K642, K69 |
| P75085 | Mpn172 | RplN | K249, K572 |
| P75087 | Mpn173 | RplX | K85, K691 |
| P75088 | Mpn174 | RplE | K142, K229 |
| P75089 | Mpn175 | RplF | K6, K42, K45, K80, K129, K200, K201, K223, K257 |
| P75090 | Mpn176 | RpsE | K27, K67, K148, K408 |
| P75091 | Mpn177 | RplO | K114, K554 |
| P75092 | Mpn178 | RplF | K3, K6, K102, K150, K178, K292 |
| P75093 | Mpn179 | RplO | K106, K146, K178, K191, K194, K56, K75, K82 |
| P75094 | Mpn180 | SecY | K182, K202, K204 |
| P75095 | Mpn181 | InfA | K504 |
| P75096 | Mpn182 | RpsM | K475 |
| P75098 | Mpn183 | RpoA | K249, K294 |
| P75100 | Mpn184 | RplQ | K107 |
| P75104 | Mpn187 | CbiO1 | K184, K261 |
| P75105 | Mpn189 | PepF | K1214, K1708 |
| P75106 | Mpn191 | PtsG | K1568, K1704, K695, K792, K81, K781, K391 |
| P75107 | Mpn192 | RpsB | K1095, K117, K1266, K1330, K1342, K1405, K1501, K1574, K501, K522, K551, K769, K863, K1693, K1700 |
| P75109 | Mpn193 | SecA | K98 |
| P75110 | Mpn197 | UvrB | K21, K50, K154, K197, K307 |
| P75112 | Mpn199 | OppC | K137, K119 |
| P75113 | Mpn200 | OppF | K100, K111, K125 |
| P75114 | Mpn202 | RplA | K111, K143, K157, K25, K76 |
| P75115 | Mpn205 | FusA | K133, K151, K239, K299, K38 |
| P75118 | Mpn207 | Ssb | K20, K165 |
| P75119 | Mpn208 | RpsR | K519, K546, K556, K568, K568, K630, K680, K278, K52 |
| P75120 | Mpn210 | RplI | K31, K44 |
| P75121 | Mpn211 | DnaB | K23, K147 |
| P75122 | Mpn212 | GatA | K28, K333 |
| P75124 | Mpn214 | Mpn214 | K12, K285 |
| P75125 | Mpn215 | Rnr | K154 |
| P75127 | Mpn216 | Def | K107, K120, K130, K147, K213, K225, K238, K35, K54, K55, K772 |
| P75128 | Mpn217 | Pgi | K16, K71, K234, K393 |
| P75129 | Mpn218 | AsnS | K292 |
| P75131 | Mpn219 | Mpn219 | K221 |
| P75132 | Mpn220 | Mpn220 | K65 |
| P75133 | Mpn221 | TopA | K36 |
| P75137 | Mpn223 | Mpn223 | K520 |
| P75144 | Mpn225 | TrxA | K6, K22, K44, K60, K64 |
| P75145 | Mpn226 | TrpS | K271, K287 |
| P75159 | Mpn227 | Mpn227 | K10, K15, K19, K22, K29, K38, K42, K82, K108, K121, K125, K155, K159, K172, K241, K285, K315, K339, K340, K376, K5, K52, K93, K462, K759, K791 |
| P75161 | Mpn228 | Mpn228 | K285, K492 |
| P75165 | Mpn229 | PpnK | K13, K125, K146, K158 |
| P75167 | Mpn230 | Mpn230 | K263, K285 |
| P75168 | Mpn231 | Mpn231 | K77, K85 |
| P75170 | Mpn232 | Mpn232 | K3, K131 |
| P75172 | Mpn233 | Mpn233 | K8, K82 |
| P75173 | Mpn234 | Mpn234 | K10, K15, K19, K22, K29, K38, K121, K125 |
| P75174 | Mpn235 | Mpn235 | K5, K37, K52, K57, K91, K101, K101, K285, K312 |
| P75175 | Mpn236 | Mpn236 | K142, K156, K325 |
| P75176 | Mpn237 | Mpn237 | K25, K40, K69, K99 |
| P75177 | Mpn239 | Mpn239 | K133, K141, K216, K228, K284, K309, K325, K330, K340, K353 |
| P75178 | Mpn240 | Mpn240 | K3, K4 |
| P75179 | Mpn241 | Mpn241 | K347, K369 |
| P75183 | Mpn243 | Rnr | K38, K74, K167 |
| P75185 | Mpn245 | Def | K5, K374, K52, K263, K285, K306 |
| P75186 | Mpn246 | Pgi | K5, K52, K177, K184, K306, K306 |
| P75187 | Mpn248 | Mpn248 | K243, K57, K83 |
| P75188 | Mpn249 | AsnS | K6 |
| P75189 | Mpn250 | Mpn250 | K11, K226, K230, K263, K295, K38, K101, K133, K161, K171, K22 |
| P75191 | Mpn252 | TopA | K5, K11, K145, K36 |
| P75192 | Mpn255 | Mpn255 | K126, K174, K201, K221, K268, K328, K502, K502, K530, K533, K92 |
| P75193 | Mpn261 | TrxA | K105, K187, K252, K360, K381, K49, K96, K306, K521, K3 |
| P75195 | Mpn262 | Mpn262 | K8, K68 |
| P75196 | Mpn263 | TrpS | K65 |
| P75197 | Mpn264 | Mpn264 | K65, K117 |
| P75198 | Mpn265 | Mpn265 | K30, K146 |
| P75199 | Mpn266 | Mpn266 | K241 |
| P75200 | Mpn267 | PpnK | K563 |
| P75201 | Mpn268 | Mpn268 | K207, K249, K559 |
| P75205 | Mpn269 | Mpn269 | K240 |
| P75206 | Mpn270 | Mpn270 | K10, K11, K121, K137, K140, K150, K215, K217, K28, K348, K35, K4, K54 |
| P75210 | Mpn271 | Mpn271 | K10, K120 |
| P75211 | Mpn273 | Mpn273 | K215, K263, K945 |
| P75212 | Mpn274 | Mpn274 | K1006, K530, K536, K543, K549, K592, K666, K806, K875, K971 |
| P75214 | Mpn275 | Mpn275 | K536, K549 |
| P75215 | Mpn276 | Mpn276 | K110, K112, K114, K138, K170, K238, K30, K31, K72, K93, K96 |
| P75216 | Mpn279 | Mpn279 | K439 |
| P75217 | Mpn280 | Mpn280 | K10, K273 |
| P75218 | Mpn282 | Mpn282 | K273, K72, K825, K872, K993, K1058, K1060 |
| P75219 | Mpn285 | Mpn285 | K256, K318, K996 |
| P75220 | Mpn287 | Mpn287 | K100, K138, K37 |
| P75221 | Mpn288 | Mpn288 | K16, K173, K19, K48 |
| P75222 | Mpn289 | Mpn289 | K106, K130, K150, K164, K173, K19 |
| P75223 | Mpn290 | Mpn290 | K16, K48, K55, K60 |
| P75225 | Mpn291 | Mpn291 | K191, K286, K368, K417, K561, K684, K28, K323 |
| P75226 | Mpn292 | Mpn292 | K169 |
| P75227 | Mpn293 | Mpn293 | K191 |
| P75228 | Mpn294 | Mpn294 | K266, K353 |
| P75229 | Mpn295 | Mpn295 | K123, K366 |
| P75230 | Mpn296 | Mpn296 | K50 |
| P75231 | Mpn297 | Mpn297 | K285 |
| P75232 | Mpn298 | Mpn298 | K67 |
| P75233 | Mpn299 | Mpn299 | K86, K91, K125 |
| P75234 | Mpn300 | Mpn300 | K17, K101 |
| P75235 | Mpn302 | CbiO1 | K101, K37 |
| P75236 | Mpn303 | PepF | K312 |
| P75237 | Mpn304 | Mpn304 | K285 |
| P75238 | Mpn305 | PtsG | K492 |
| P75239 | Mpn306 | RpsB | K13, K158, K285, K263, K285 |
| P75240 | Mpn308 | UvrB | K77, K85 |
| P75245 | Mpn309 | OppC | K3, K6, K8, K9, K23, K33, K65, K136, K178, K205, K220, K240, K266, K308, K362, K393, K425, K455, K466, K502, K521, K70 |
| P75246 | Mpn310 | Hmw2 | K3, K108, K111, K121, K140, K236, K342, K67 |
| P75247 | Mpn312 | Mpn312 | K4, K122, K13 |
| P75248 | Mpn313 | Mpn313 | K162, K272, K292, K363, K98 |
| P75249 | Mpn314 | MraZ | K6, K68, K945 |
| P75250 | Mpn315 | MraW | K3, K65, K81, K83, K87, K96, K117, K121, K174, K201, K268, K308 |
| P75252 | Mpn316 | ThyA | K28, K46, K88, K134, K143, K219 |
| P75257 | Mpn317 | Mpn317 | K60, K66, K74 |
| P75258 | Mpn318 | FolA | K74, K267 |
| P75264 | Mpn320 | NrdF | K574, K290, K290, K567, K567, K308,K310 |
| P75268 | M-1 | M-1 | K284 |
| P75269 | Mpn321 | NrdI | K112, K166, K279, K504, K537, K541, K565, K91 |
| P75270 | Mpn322 | NrdE | K310, K504, K537 |
| P75271 | Mpn323 | RplU | K142, K331, K493, K486, K528, K549, K873, K876, K820, K5 |
| P75275 | Mpn324 | Mpn324 | K345 |
| P75288 | Mpn325 | Mpn325 | K4, K56 |
| P75289 | Mpn328 | Tig | K224, K349, K416, K440 |
| P75292 | Mpn330 | Lon | K48, K73 |
| P75293 | Mpn331 | Mpn331 | K536, K549 |
| P75294 | Mpn332 | Mpn332 | K1006, K530, K536, K592, K666, K806, K875, K971 |
| P75298 | Mpn336 | Mpn336 | K96, K110, K112, K114, K138, K170, K238, K30, K31, K72 |
| P75302 | Mpn337 | Mpn337 | K273 |
| P75303 | Mpn338 | Mpn338 | K10, K273, K72, K825, K872, K993, K1058, K1060 |
| P75304 | Mpn348 | RpoD | K256, K318, K996 |
| P75305 | Mpn349 | DnaG | K37, K100, K138 |
| P75306 | Mpn352 | GlyQS | K16, K19, K48, K173 |
| P75308 | Mpn353 | Mpn353 | K19, K60, K106, K130, K150, K164, K173 |
| P75309 | Mpn354 | RpmE | K16, K48, K55 |
| P75310 | Mpn359 | PrfA | K191, K286, K28, K323, K368, K417, K561, K684, K169 |
| P75312 | Mpn360 | Mpn360 | K191 |
| P75313 | Mpn361 | Mpn361 | K266, K353, K123, K366, K50 |
| P75315 | Mpn362 | Mpn362 | K285 |
| P75317 | Mpn365 | Mpn365 | K67 |
| P75322 | Mpn372 | Mpn372 | K86, K91, K125 |
| P75343 | Mpn377 | Mpn377 | K17, K101 |
| P75344 | Mpn379 | PolA | K37, K101, K125, K146, K158, K263, K285, K285, K77, K85 |
| P75349 | Mpn381 | Mpn381 | K3, K131 |
| P75350 | Mpn384 | LeuS | K8, K82 |
| P75351 | Mpn386 | Mpn386 | K10, K15, K19, K22, K29, K38, K121, K125 |
| P75352 | Mpn387 | Mpn387 | K5, K37, K52, K57, K91, K101, K101, K285, K312 |
| P75353 | Mpn388 | LplA | K142, K156, K25, K325, K40, K69, K99 |
| P75354 | Mpn389 | PdhD | K133, K141, K216, K228, K284, K309, K325, K330, K340, K353 |
| P75355 | Mpn390 | PdhC | K3, K4 |
| P75358 | Mpn391 | PdhB | K74, K167, K38, K347, K369 |
| P75359 | Mpn392 | PdhA | K5, K52, K263, K285, K306, K374, K306 |
| P75360 | Mpn393 | Nox | K5, K52, K177, K184, K306 |
| P75361 | Mpn394 | SpoT | K6, K83, K226, K57 |
| P75362 | Mpn395 | GreA | K668 |
| P75363 | Mpn396 | Mpn396 | K315 |
| P75364 | Mpn397 | ProS | K692 |
| P75365 | Mpn398 | AlaS | K419, K606 |
| P75366 | Mpn399 | MnmA | K23, K9 |
| P75367 | Mpn401 | FtsY | K70, K81, K308 |
| P75368 | Mpn402 | P115 | K161, K196, K200, K206, K298, K304, K342 |
| P75382 | Mpn419 | Pta | K111, K67 |
| P75385 | Mpn422 | Pgk | K108, K121 |
| P75386 | Mpn425 | GapA | K236 |
| P75388 | Mpn426 | DnaK | K37, K56, K96 |
| P75389 | Mpn428 | Hmw1 | K80, K90, K144, K178, K184, K194, K244, K256, K281, K325, K446 |
| P75390 | Mpn429 | Hmw3 | K24, K46, K95, K166, K231, K240, K245, K378, K507, K543, K549, K554, K573, K587, K775 |
| P75391 | Mpn430 | Mpn430 | K10, K19, K74, K117, K119, K133, K148, K173, K177, K193, K201, K229, K231, K249, K266, K270, K285, K297, K303, K339, K370, K415, K461, K492, K499, K526, K541, K628, K669 |
| P75392 | Mpn434 | Mpn434 | K2, K11, K12, K14, K15, K22, K27, K43, K56, K63, K82, K97, K100, K123, K130, K165, K279 |
| P75393 | Mpn447 | PepP | K21, K24, K28, K35, K38, K48, K53, K65, K84, K96, K100, K103, K115, K120, K124, K128, K132, K140, K155, K157, K162, K164, K172, K220, K225, K247, K259, K269, K314, K336, K346 |
| P75394 | Mpn452 | RpmG1 | K10, K26, K28, K36, K38, K41, K52, K57, K86, K92, K98, K107, K108, K122 |
| P75395 | Mpn453 | Mpn453 | K8, K10, K17, K19, K22, K43, K45, K49, K63, K64, K75, K92, K110, K150, K159, K224, K234, K255, K260, K278, K302, K320, K330, K346, K388 |
| P75396 | Mpn461 | Cmk | K2, K18, K66, K106, K135, K141 |
| P75398 | Mpn470 | AzoR | K21, K28, K30, K45, K76, K87, K93, K99, K101, K113, K115, K117, K132, K133, K144, K154, K159, K161, K173 |
| P75399 | Mpn471 | ValS | K353, K630, K701, K822, K848 |
| P75401 | Mpn474 | UlaE | K131, K223, K225, K253, K259, K261, K303, K312, K333, K359, K363, K428 |
| P75402 | Mpn475 | UlaB | K187, K220, K225, K253, K290 |
| P75403 | Mpn476 | Cmk | K11, K78, K104 |
| P75404 | Mpn478 | AzoR | K3, K20, K36, K42, K49, K55 |
| P75405 | Mpn479 | ValS | K23, K34, K155, K167, K192, K205, K213 |
| P75409 | Mpn480 | UlaE | K26, K44, K52, K76, K132, K176 |
| P75419 | Mpn492 | RpoC | K23, K62 |
| P75420 | Mpn495 | RpoB | K14, K35, K118, K184, K352, K760, K960 |
| P75421 | Mpn499 | Mpn499 | K14, K65, K204, K244, K290 |
| P75422 | Mpn502 | Mpn502 | K21 |
| P75423 | Mpn506 | Mpn506 | K141, K158, K189, K209, K219 |
| P75424 | M-1 | M-1 | K10, K52 |
| P75425 | Mpn515 | RpoC | K27, K37, K40, K115, K122, K136, K179, K270, K344, K345 |
| P75426 | Mpn516 | RpoB | K18, K19, K21, K22, K32, K34, K46, K55, K58, K133, K155 |
| P75429 | Mpn517 | Mpn517 | K5, K7, K9, K21, K42, K49, K74, K178, K232, K234, K241 |
| P75430 | M-1 | M-1 | K26, K173 |
| P75434 | Mpn518 | Mpn518 | K126, K229 |
| P75437 | Mpn521 | Mpn521 | K114 |
| P75438 | Mpn526 | Ppa | K116, K128 |
| P75439 | Mpn528 | Mpn528 | K4, K11, K22, K34, K42, K45, K76, K155 |
| P75440 | Mpn529 | Mpn529 | K101, K105, K132, K152, K194, K238, K248, K252, K354 |
| P75441 | Mpn530 | ClpB | K6, K21, K77, K181, K203, K204, K359 |
| P75442 | Mpn531 | AckA | K23, K119, K136, K164, K213, K290, K329 |
| P75445 | M-1 | M-1 | K152 |
| P75446 | Mpn533 | RplJ | K3, K29, K31 |
| P75453 | Mpn534 | RplL | K21, K26, K31, K41, K42, K47, K56, K57, K59, K63, K68, K69, K75, K79 |
| P75454 | Mpn535 | RpsT | K2, K11, K14, K15, K19, K20, K24, K25, K26, K34, K40, K41, K51, K55, K59, K66, K77, K86, K87, K96 |
| P75455 | Mpn538 | Rnc | K14, K34, K38, K51 |
| P75456 | Mpn539 | PlsX | K45, K50 |
| P75457 | Mpn541 | Mpn541 | K5, K34, K47, K53, K65, K102, K116, K136 |
| P75458 | Mpn542 | Mpn542 | K356 |
| P75459 | Mpn543 | ThrS | K356, K364, K388 |
| P75460 | Mpn544 | Mpn544 | K425, K446, K460, K472, K511, K643 |
| P75461 | Mpn545 | ArgS | K28, K33, K51, K59, K72, K105, K118, K159, K229, K232, K463 |
| P75465 | Mpn546 | RsmG | K142 |
| P75466 | Mpn547 | Obg | K20, K129, K226, K229, K230, K270, K296, K302, K325, K333, K347 |
| P75467 | Mpn553 | PepA | K17, K18, K57, K62, K63, K99, K130, K150 |
| P75469 | Mpn555 | GroL | K9, K82, K203, K228 |
| P75470 | Mpn556 | GroS | K5, K13, K14, K27, K40, K80 |
| P75471 | M-1 | M-1 | K2, K3, K6, K9, K12, K13, K20, K23, K24, K25, K27, K28, K32, K34, K35, K37, K38, K41, K42, K43, K44, K46, K47, K49, K50, K52, K53, K55, K56, K58, K60, K61, K63, K64, K65, K67, K69, K71, K72, K74, K75, K76, K77, K78, K80, K81, K82, K84, K86, K87, K88, K89, K90, K91, K92, K93, K95, K96, K97, K98, K101, K102, K103, K104, K105, K106, K107, K108, K110, K111, K112, K114, K115, K116, K117, K119, K120, K121, K123, K124, K125, K126, K128, K129, K132, K133, K134, K135, K136, K137, K138, K140, K141, K142 |
| P75476 | Mpn559 | GlyA | K69, K148, K191, K201, K230, K341 |
| P75477 | Mpn561 | AtpD | K73, K176, K182, K267, K364, K432 |
| P75478 | Mpn562 | AtpG | K19, K93, K101, K142, K145, K171, K252 |
| P75479 | Mpn563 | AtpA | K134, K177 |
| P75481 | Mpn564 | AtpH | K6, K22, K29, K37, K38, K42, K67, K126, K295 |
| P75482 | Mpn565 | AtpF | K4, K18, K88, K92, K125, K134, K156, K254, K255, K282, K310 |
| P75483 | Mpn566 | Eno | K274, K406 |
| P75485 | Mpn567 | Mpn567 | K3, K29, K38, K54, K83, K501 |
| P75486 | Mpn568 | PstB | K6, K8 |
| P75489 | Mpn572 | PstA | K698 |
| P75494 | Mpn573 | GroL | K8 |
| P75496 | Mpn574 | GroS | K242 |
| P75497 | Mpn575 | RpsI | K3, K221, K83, K87, K96, K117, K121, K174, K201, K268 |
| P75498 | M-1 | M-1 | K134, K30 |
| P75499 | Mpn576 | RpsI | K30, K88, K46 |
| P75500 | Mpn577 | UvrA | K28 |
| P75502 | Mpn578 | Mpn578 | K219, K143 |
| P75504 | Mpn584 | Mpn584 | K60, K66 |
| P75506 | Mpn588 | Mpn588 | K74, K74 |
| P75508 | Mpn590 | Mpn590 | K267 |
| P75509 | Mpn591 | Mpn591 | K62, K84 |
| P75510 | Mpn592 | Mpn592 | K5, K38, K52, K285, K290, K290, K306, K567, K567, K574, K308,K310, K284 |
| P75511 | M-1 | M-1 | K541 |
| P75512 | Mpn595 | Mpn595 | K91, K112, K166, K279, K504, K537, K565 |
| P75513 | Mpn596 | Mpn596 | K310, K504, K537 |
| P75514 | Mpn598 | Mpn598 | K5, K142, K331, K493, K486, K528, K549, K820, K873, K876, K345 |
| P75515 | Mpn599 | Mpn599 | K4, K56 |
| P75516 | Mpn600 | Mpn600 | K73, K224, K349, K416, K440 |
| P75517 | Mpn601 | Mpn601 | K48 |
| P75518 | Mpn602 | Mpn602 | K536, K549 |
| P75519 | Mpn606 | Mpn606 | K592, K666, K806, K875, K971, K530, K536, K1006 |
| P75521 | Mpn608 | Mpn608 | K238, K30, K31, K72, K93, K96, K110, K112, K114, K138, K170 |
| P75522 | Mpn609 | PstB | K273 |
| P75524 | Mpn610 | PstA | K10, K72, K273, K825, K872, K993, K1058, K1060 |
| P75525 | Mpn616 | RpsI | K256, K318, K996 |
| P75526 | Mpn619 | UvrA | K37, K100, K138 |
| P75527 | Mpn621 | Mpn621 | K16, K19, K48, K173, K106, K130, K150, K164, K19 |
| P75528 | Mpn622 | RpsO | K16, K48, K55, K60 |
| P75529 | Mpn625 | Mpn625 | K28, K169, K191, K286, K323, K368, K417, K561, K684 |
| P75530 | Mpn627 | PtsI | K191 |
| P75531 | Mpn628 | GpmI | K123, K266, K353, K366, K50 |
| P75532 | Mpn629 | TpiA | K285 |
| P75533 | Mpn631 | Tsf | K67, K86, K91, K125, K17, K101 |
| P75534 | Mpn636 | Frr | K37, K101, K312, K285 |
| P75535 | Mpn638 | Mpn638 | K492 |
| P75539 | Mpn640 | Mpn640 | K13, K125, K146, K158, K263, K285 |
| P75540 | Mpn642 | Mpn642 | K77, K85, K339, K131, K3 |
| P75541 | Mpn643 | Mpn643 | K8, K82 |
| P75542 | Mpn644 | Mpn644 | K10, K15, K19, K22, K29, K38, K121, K125, K159, K172, K241, K285, K315, K340, K376 |
| P75543 | Mpn645 | Mpn645 | K5, K52, K57, K91, K101, K101, K285, K312 |
| P75544 | Mpn646 | Mpn646 | K25, K40, K69, K99, K133, K141, K156, K216, K228, K284, K309, K325, K330, K340, K353 |
| P75545 | Mpn647 | Mpn647 | K3, K4 |
| P75546 | Mpn649 | Mpn649 | K347, K369 |
| P75548 | Mpn650 | Mpn650 | K38, K74, K167 |
| P75549 | Mpn660 | Mpn660 | K5, K52, K263, K285, K306, K374 |
| P75550 | Mpn662 | RpsP | K5, K177, K184, K306, K306, K52 |
| P75551 | Mpn664 | MsrB | K6, K57, K83, K226 |
| P75552 | Mpn665 | Tuf | K668, K315, K692 |
| P75553 | Mpn666 | Mpn666 | K419, K606 |
| P75554 | Mpn667 | GalU | K23, K9 |
| P75555 | Mpn668 | Mpn668 | K70, K81, K308 |
| P75556 | Mpn669 | Mpn669 | K161, K196, K200, K206, K298, K304, K342 |
| P75558 | Mpn670 | Mpn670 | K13, K28, K36, K50, K65, K67, K111, K116, K120, K194, K197 |
| P75559 | Mpn671 | FtsH | K9, K16, K27, K28, K45, K80, K98, K112, K133, K205, K249 |
| P75560 | Mpn673 | Ldh | K16, K107, K110, K127 |
| P75563 | Mpn674 | Mpn674 | K24, K45, K102, K231, K275, K372, K368, K462, K496 |
| P75564 | Mpn676 | Mpn676 | K6, K24, K107, K197, K687 |
| P75569 | Mpn677 | Mpn677 | K4, K11, K16, K16, K23, K27, K31, K110, K112, K114, K136, K139, K147, K213, K229, K256, K299, K333, K353 |
| P75575 | Mpn678 | GltX | K69, K142, K202, K426 |
| P75576 | Mpn686 | DnaA | K686, K96, K642 |
| P75577 | Mpn688 | Mpn688 | K69 |
| Q11132 | Mpn690 | Mpn690 | K43, K45 |
| Q50289 | Mpn700 | Mpn700 | K772 |
| Q50291 | Mpn702 | Mpn702 | K130 |
| Q50293 | Mpn704 | Mpn704 | K229, K238, K35, K55 |
| Q50294 | Mpn705 | Mpn705 | K120, K213, K229, K54 |
| Q50295 | Mpn706 | Mpn706 | K16, K54, K71, K147, K234, K292, K393 |
| Q50296 | Mpn707 | Mpn707 | K221, K65 |
| Q50297 | Mpn708 | Mpn708 | K36, K520, K60, K64 |
| Q50298 | Mpn709 | Mpn709 | K22, K8 |
| Q50299 | Mpn710 | Mpn710 | K271, K287, K462 |
| Q50300 | Mpn711 | Mpn711 | K759, K791, K92 |
| Q50301 | Mpn712 | Mpn712 | K37, K220 |
| Q50302 | Mpn713 | Mpn713 | K174, K178, K182 |
| Q50303 | Mpn714 | Mpn714 | K49, K187, K225, K267, K308 |
| Q50305 | Mpn716 | Mpn716 | K145 |
| Q50306 | Mpn717 | Mpn717 | K112, K134, K145, K165, K171, K252 |
| Q50307 | Mpn718 | Mpn718 | K96, K177, K184, K230, K328 |
| Q50308 | Mpn719 | Mpn719 | K105 |
| Q50309 | Mpn720 | Mpn720 | K92, K126, K360 |
| Q50310 | Mpn721 | Mpn721 | K6, K29, K38, K387 |
| Q50312 | Mpn723 | Mpn723 | K295, K310, K8, K18 |
| Q50313 | Mpn724 | Mpn724 | K121, K125, K202, K255, K274, K282, K340, K38, K5, K52, K8, K9 |
| Q50314 | Mpn725 | Mpn725 | K143, K165, K241, K25, K54, K83, K93, K156, K310, K406 |
| Q50315 | Mpn726 | Mpn726 | K501, K780 |
| Q50316 | Mpn727 | Mpn727 | K6, K117 |
| Q50327 | Mpn738 | Mpn738 | K698 |
| Q50328 | Mpn739 | Mpn739 | K8, K242 |
| Q50329 | M-1 | M-1 | K221, K83, K87, K96, K117, K121, K174, K201, K268 |
| Q50330 | Mpn740 | Mpn740 | K3, K134, K30 |
| Q50331 | Mpn741 | Mpn741 | K30, K46, K88, K143, K219 |
| Q50332 | Mpn742 | Mpn742 | K28 |
| Q50334 | Mpn744 | Mpn744 | K66, K60 |
| Q50341 | Mpn751 | Mpn751 | K74 |
| Q50360 | Mpn769 | Mpn769 | K267, K574, K290, K290, K567 |
| Q50362 | Mpn771 | Mpn771 | K284 |
| Q50363 | Mpn772 | Mpn772 | K112, K166, K279, K504, K537, K541, K565, K91 |
| Q50365 | Mpn774 | Mpn774 | K310, K504, K537, K331, K493, K486, K528, K549, K820, K873, K876, K5 |
| Q59547 | Mpn796 | Mpn796 | K345, K4, K56 |
| Q59549 | Mpn798 | Mpn798 | K73, K224, K349, K416, K440, K48, K536, K549 |
| Q9EXC9 | Mpn799 | Mpn799 | K1006, K530, K536, K592, K666 |
| Q9EXD2 | Mpn802 | Mpn802 | K806 |
| Q9EXD3 | Mpn803 | Mpn803 | K30, K31, K72, K93, K96, K110, K112, K114, K138, K170, K238 |
| Q9EXD4 | Mpn804 | Mpn804 | K273 |
| Q9EXD7 | Mpn807 | Mpn807 | K10, K72, K273 |
| Q9EXD8 | Mpn808 | Mpn808 | K256, K318, K825, K872, K993, K996, K1058, K1060 |
| P13927 | MG_001 | DNA polymerase III, beta subunit | K2, K6, K31, K48, K52, K55, K78, K101, K129, K135, K166, K172, K204, K221, K229, K234, K250, K256, K263, K269, K311, K320, K335, K340, K346, K415 |
| P20796 | MG_002 | DNA gyrase subunit B | K308, K335, K353, K366, K475, K485, K520, K554, K566, K579, K632, K682, K693, K694, K706, K716 |
| P22746 | MG_003 | DNA gyrase subunit A | K286, K287, K471, K511, K618, K662, K736, K748, K766, K775 |
| P22747 | MG_004 | Serine--tRNA ligase | K2, K16, K29, K36, K51, K244, K299, K345, K348, K415, K463 |
| P35888 | MG_005 | Ribose-phosphate pyrophosphokinase | K11, K30, K37, K75, K169, K213, K217, K218, K243 |
| P36255 | MG_006 | Putative DNA polymerase I | K286, K388, K473, K513, K600, K617, K628, K694, K710, K826 |
| P36263 | MG_007 | 50S ribosomal protein L10 | K29, K40, K66, K98, K139, K140, K152, K164, K172 |
| P47246 | MG_010 | Elongation factor G | K3, K14, K17, K22, K27, K43, K50, K52, K60, K82, K123, K133, K151, K163, K204, K226, K239, K259, K262, K288, K299, K312, K325, K366, K373, K385, K519, K531, K598, K601, K612, K619, K626, K628, K664, K669, K694 |
| P47247 | MG_011 | Elongation factor Tu | K4, K6, K10, K17, K18, K28, K31, K42, K140, K209, K344, K357 |
| P47248 | MG_012 | 50S ribosomal protein L7/L12 | K2, K5, K11, K26, K54, K62, K66, K78, K81, K93, K120 |
| P47249 | MG_013 | Enolase | K3, K27, K56, K95, K135, K157, K183, K222, K232, K237, K261, K274, K304, K322, K347, K376, K397, K406, K424 |
| P47250 | MG_014 | Phosphoglycerate mutase | K2, K5, K12, K20, K38, K40, K41, K42, K47, K71, K74, K75, K85, K88, K93, K96, K100, K105, K114, K132, K164, K172, K212, K214, K220, K240, K253 |
| P47251 | MG_015 | Pyruvate kinase | K25, K47, K77, K86, K96, K111, K113, K130, K134, K145, K238, K269, K280, K327, K371, K410, K421, K426, K431, K446, K450, K458, K496 |
| P47252 | MG_016 | Lactate dehydrogenase | K6, K8, K16, K24, K45, K102, K110, K127, K231, K275, K368, K372 |
| P47253 | MG_017 | Trigger factor | K224, K349, K416, K440 |
| P47254 | MG_018 | DNA-directed RNA polymerase subunit beta | K18, K19, K21, K22, K32, K34, K46, K55, K58, K133, K155 |
| P47255 | MG_019 | DNA-directed RNA polymerase subunit beta' | K5, K7, K9, K21, K42, K49, K74, K178, K232, K234, K241 |
| P47256 | MG_020 | Tryptophanyl-tRNA synthetase | K65 |
| P47258 | MG_021 | DnaK protein | K7, K37, K56, K69, K96, K558 |
| P47259 | MG_022 | DnaJ protein | K2, K24, K32, K257, K300, K334, K378, K385 |
| P47260 | MG_023 | GrpE protein | K3, K35, K44, K68, K78, K109, K135, K147, K150, K151, K158, K161, K169, K173, K182, K195 |
| P47261 | MG_024 | Chaperonin GroEL | K3, K9, K82, K90, K135, K193, K203, K228, K314, K327, K393, K451, K484, K525 |
| P47264 | MG_025 | Fructose-bisphosphate aldolase | K24, K54, K65, K67, K75, K84, K90, K106, K113, K116, K120, K124, K130, K137, K140, K156, K165, K179, K193, K195, K197, K201, K202, K203, K207, K208, K237, K239, K248, K256, K259, K269, K270, K285, K286, K289, K290, K291, K303 |
| P47265 | MG_026 | Triosephosphate isomerase | K4, K6, K12, K33, K56, K77, K86, K103, K126, K130, K135, K138, K164, K199, K238 |
| P47266 | MG_027 | Glyceraldehyde-3-phosphate dehydrogenase | K4, K32, K39, K182, K190, K211, K246, K286, K296, K308, K320 |
| P47267 | MG_028 | Phosphoglycerate kinase | K121, K130, K137, K162, K191, K214, K223, K248, K306, K378 |
| P47268 | MG_029 | Elongation factor P | K8, K21, K31, K41, K48, K92, K141, K152, K178, K180 |
| P47269 | MG_030 | Uncharacterized protein MG_030 | K28, K36, K55, K76, K87, K98, K102, K106, K118, K141, K142, K147, K156, K210, K212, K224 |
| P47270 | MG_031 | 50S ribosomal protein L2 | K3, K134, K148, K157, K231 |
| P47272 | MG_034 | DNA polymerase III, alpha chain | K105, K340, K388, K888, K1009 |
| P47273 | MG_035 | DNA polymerase III, epsilon chain | K30, K76, K133 |
| P47274 | MG_036 | DNA ligase | K67, K117 |
| P47275 | MG_037 | Aspartate--tRNA ligase | K25, K29, K74, K83, K99, K168, K210, K218, K281, K554 |
| P47276 | MG_038 | Phenylalanine--tRNA ligase, beta subunit | K69, K86, K142, K179, K185, K203 |
| P47277 | MG_039 | Phenylalanine--tRNA ligase, alpha subunit | K7, K9, K24, K39, K40, K65, K89, K93, K109, K126, K133, K160, K180, K186, K191, K208, K217, K240, K256, K267, K273, K278, K279, K281, K284, K296, K304, K308, K317, K328, K334, K339, K342, K344, K353, K356, K361, K366, K367 |
| P47278 | MG_040 | Peptidyl-tRNA hydrolase | K11, K20, K36, K46, K80, K87, K100, K106, K181 |
| P47280 | MG_041 | Thioredoxin | K6, K22, K44, K60, K64, K71, K86, K93, K104 |
| P47281 | MG_042 | Methionyl-tRNA formyltransferase | K3, K9, K28, K62, K101, K103, K107, K111, K120, K213, K225, K238, K240 |
| P47282 | MG_044 | 50S ribosomal protein L6 | K3, K6, K42, K45, K80, K129, K133, K150, K151, K155, K166, K178, K182, K187, K196, K198, K200, K201, K223, K257 |
| P47283 | MG_045 | 50S ribosomal protein L18 | K23, K25, K38, K48, K49, K50, K51, K55, K67, K70, K81, K82, K106, K110, K123 |
| P47284 | MG_046 | 50S ribosomal protein L5 | K3, K6, K7, K11, K24, K46, K68, K75, K102, K130, K145, K150, K178, K202 |
| P47285 | MG_047 | 30S ribosomal protein S5 | K3, K5, K9, K18, K25, K27, K67, K148, K163 |
| P47287 | MG_048 | Transcription elongation factor GreA | K11, K18, K21, K41, K64, K111, K139 |
| P47288 | MG_049 | Uncharacterized protein MG_049 | K107, K125, K149, K180 |
| P47292 | MG_053 | DNA helicase II | K3, K28, K33, K55, K164, K245, K624 |
| P47294 | MG_054 | Ribose-5-phosphate isomerase A | K8, K21, K24, K28, K42, K44, K50, K79, K90, K109, K110, K121, K129, K132, K142, K160 |
| P47295 | MG_056 | Glucose-6-phosphate isomerase | K5, K16, K52, K71, K177, K184, K234, K306, K309, K366, K393, K458, K471 |
| P47296 | MG_057 | 2-deoxyribose-5-phosphate aldolase | K2, K35, K44, K137, K141, K163, K179, K182 |
| P47297 | MG_058 | Phosphoribosyl pyrophosphate synthetase | K4, K6, K8, K12, K15, K18, K23, K24, K35, K36, K146, K224, K241, K269, K289, K290, K293 |
| P47299 | MG_059 | Purine-nucleoside phosphorylase | K2, K3, K4, K5, K14, K36, K37, K43, K59, K71, K87, K91, K99, K103, K119, K120, K148, K161, K170, K184, K201, K213, K215, K216, K219 |
| P47300 | MG_061 | Histidyl-tRNA synthetase | K11, K25, K27, K71, K78, K106, K107, K172, K203, K299, K312 |
| P47301 | MG_062 | Threonyl-tRNA synthetase | K425, K446, K460, K472, K511, K643 |
| P47302 | MG_063 | Threonine-tRNA ligase ThrS | K28, K33, K51, K59, K72, K105, K118, K159, K229, K232, K463 |
| P47303 | MG_064 | Aspartyl-tRNA synthetase | K142 |
| P47304 | MG_065 | Ribonuclease R | K20, K129, K226, K229, K230, K270, K296, K302, K325, K333, K347 |
| P47305 | MG_066 | DNA-directed RNA polymerase subunit alpha | K249, K294 |
| P47306 | MG_067 | 30S ribosomal protein S2 | K24, K63, K98, K101, K112, K147 |
| P47307 | MG_069 | Phosphoenolpyruvate-protein phosphotransferase | K30, K65, K84 |
| P47308 | MG_070 | HPr kinase/phosphorylase | K112, K170, K200, K238, K285, K295 |
| P47310 | MG_072 | Glycerol-3-phosphate dehydrogenase [NAD(P)+] | K50, K79 |
| P47311 | MG_073 | Glycerol kinase | K16, K23, K48, K129, K312, K341 |
| P47312 | MG_075 | 50S ribosomal protein L1 | K4, K7, K19, K25, K49, K53, K63, K87, K88, K93, K111, K118, K143, K157, K174, K197, K210, K218, K232, K245 |
| P47314 | MG_077 | Ribosome-recycling factor | K20, K165 |
| P47315 | MG_078 | Uncharacterized protein MG_078 | K3, K6, K45, K48, K55, K58, K68, K75, K90, K93, K95, K96, K98, K102, K150, K157, K167, K178, K216, K217, K220, K292, K353 |
| P47316 | MG_079 | 30S ribosomal protein S18 | K2, K5, K10, K16, K24, K35, K38, K40, K41, K42, K49, K55, K62, K63, K72 |
| P47317 | MG_080 | 50S ribosomal protein L15 | K3, K6, K15, K19, K25, K41, K45, K56, K75, K82, K106, K146, K178, K194 |
| P47318 | MG_081 | 50S ribosomal protein L27 | K4, K11, K12, K13, K14, K17, K18, K26, K31, K32, K34, K45, K48, K50 |
| P47319 | MG_082 | Ribosome-associated protein Y | K7, K11, K12, K14, K23, K24, K26, K28, K31, K44, K60, K63, K85 |
| P47321 | MG_084 | Translation initiation factor IF-1 | K27, K36, K42 |
| P47323 | MG_085 | 30S ribosomal protein S13 | K3, K7, K22 |
| P47324 | MG_086 | 30S ribosomal protein S4 | K46, K132 |
| P47325 | MG_087 | 30S ribosomal protein S11 | K13, K20, K23, K74 |
| P47326 | MG_088 | 50S ribosomal protein L17 | K4, K13, K16, K17, K19, K22, K24, K34, K52, K58, K67, K78, K118 |
| P47327 | MG_089 | 50S ribosomal protein L29 | K12, K20, K25, K30 |
| P47328 | MG_090 | Translation initiation factor IF-2 | K28, K64, K89, K109, K122, K237, K316, K507, K562, K565, K626, K627, K634 |
| P47329 | MG_091 | 50S ribosomal protein L20 | K2, K3, K7, K15, K16, K30, K52, K66, K95, K100, K113, K114, K118 |
| P47330 | MG_092 | Transcription termination factor Rho | K2, K7 |
| P47331 | MG_094 | 30S ribosomal protein S16 | K2, K5, K11, K14, K15 |
| P47332 | MG_095 | 30S ribosomal protein S17 | K4, K26, K77, K78 |
| P47333 | MG_096 | Peptide deformylase | K16, K23, K71, K124 |
| P47334 | MG_097 | Isoleucyl-tRNA synthetase | K29, K70, K134, K230, K793, K859, K874 |
| P47335 | MG_098 | Peptide chain release factor 1 | K3, K17, K19, K23, K24, K27, K35, K39, K42, K45, K76, K87, K93, K112, K118, K126, K128, K136, K140, K160, K162, K230, K287, K314, K334 |
| P47336 | MG_099 | Putative ATP-dependent Clp protease proteolytic subunit | K3, K6, K8, K12, K13, K20, K26, K32, K33, K35, K38, K41, K42, K52, K53 |
| P47338 | MG_101 | 60 kDa chaperonin | K9, K25, K82, K90, K135, K203 |
| P47339 | MG_102 | 10 kDa chaperonin | K3, K5, K13, K14, K27, K40, K41, K42, K49, K55, K62, K63, K72 |
| P47340 | MG_103 | Putative hydrolase | K10, K11, K28, K35, K54, K120, K240 |
| P47342 | MG_104 | Cell division protein FtsZ | K3, K24, K39, K40, K51, K82, K97, K106, K133, K138, K151, K191, K201, K321, K328 |
| P47343 | MG_105 | Cell division protein FtsA | K204, K209, K215, K388 |
| P47344 | MG_106 | 50S ribosomal protein L34 | K3, K16, K22, K24 |
| P47345 | MG_107 | Ribosomal-protein-alanine acetyltransferase | K5, K6, K8, K14, K17, K18, K21, K22, K25, K28, K29, K34, K39, K46, K50 |
| P47346 | MG_109 | Elongation factor Ts | K19, K27, K32, K50, K76, K85, K94, K104, K136, K150, K153, K188, K208, K216, K223, K224, K265, K271 |
| P47347 | MG_110 | 50S ribosomal protein L32 | K2, K3, K9, K12, K13, K15, K18, K19, K24, K26, K42, K47 |
| P47348 | MG_111 | Tyrosyl-tRNA synthetase | K11, K74, K151, K188, K201, K211, K314, K326 |
| P47349 | MG_112 | ATP-dependent Clp protease ATP-binding subunit ClpX | K359, K363, K428 |
| P47350 | MG_113 | ATP-dependent Clp protease proteolytic subunit | K3, K5, K6, K8, K12, K13, K18, K20, K26, K28, K32, K33, K35, K38, K41, K42, K53 |
| P47351 | MG_114 | ClpB protein | K6, K21 |
| P47352 | MG_115 | Fructose-bisphosphate aldolase, class I | K24, K54, K65, K67, K106, K120, K124, K137, K165, K256 |
| P47353 | MG_116 | Transketolase | K14, K37, K77, K82, K114, K525, K598, K600, K618 |
| P47354 | MG_117 | Ribulose-phosphate 3-epimerase | K20, K180, K200 |
| P47355 | MG_118 | Ribose-phosphate pyrophosphokinase | K11, K30, K37, K75 |
| P47356 | MG_119 | Transaldolase | K9, K148, K242 |
| P47357 | MG_120 | 6-phosphofructokinase | K2, K5, K11, K29, K66, K89, K106, K114, K116, K138, K140, K152, K153, K205, K213, K217, K223, K224, K232, K233, K257, K286, K326 |
| P47358 | MG_121 | Transcriptional regulator, DeoR family | K170, K416, K420 |
| P47359 | MG_122 | Thymidine phosphorylase | K2, K5, K13, K17, K27, K36, K38, K46, K88, K105, K142, K175, K187 |
| P47361 | MG_123 | ParC protein | K32, K50, K65, K79, K204, K280, K384, K416, K428 |
| P47362 | MG_124 | GyrA protein | K2, K286, K374, K376, K503, K569, K620, K662, K736, K772, K827 |
| P47363 | MG_125 | DNA topoisomerase 1 | K36 |
| P47364 | MG_126 | 50S ribosomal protein L11 | K6, K22, K32, K58, K78, K104, K113 |
| P47365 | MG_127 | Ribosomal RNA large subunit methyltransferase F | K19, K204, K235 |
| P47366 | MG_130 | 30S ribosomal protein S12 | K45, K98 |
| P47368 | MG_131 | 30S ribosomal protein S7 | K3, K4, K6, K8, K9, K10, K11, K23, K33, K39, K41, K45, K50, K63, K65, K67, K70, K90, K91, K93, K102, K108, K112, K116, K121, K125, K127, K136, K137 |
| P47369 | MG_133 | 50S ribosomal protein L3 | K6, K8, K12, K24, K46, K48, K62, K83, K92, K108, K112, K121, K130, K134, K140, K183 |
| P47370 | MG_134 | Protein-export protein SecA | K98, K137, K237, K304, K353, K651, K808 |
| P47372 | MG_135 | Adenylate kinase | K7, K11, K24, K30, K34, K55, K156, K184, K191 |
| P47373 | MG_136 | 30S ribosomal protein S1 | K11, K14, K19, K33, K34, K35, K38, K53, K56, K80, K97, K101, K119, K137, K149, K150, K152 |
| P47374 | MG_137 | Uncharacterized protein MG_137 | K10, K11, K17, K22, K25, K28, K31 |
| P47376 | MG_138 | DNA primase | K37, K77, K100, K119, K138, K213, K226, K234, K257, K262, K266, K299, K300 |
| P47378 | MG_139 | 30S ribosomal protein S6 | K16, K23, K72, K98, K107 |
| P47380 | MG_140 | 50S ribosomal protein L9 | K10, K16, K38, K41, K72, K111, K124 |
| P47381 | MG_141 | 30S ribosomal protein S13 | K3, K7, K22 |
| P47382 | MG_142 | 50S ribosomal protein L33 | K2, K3, K6, K8, K11, K12, K24, K31, K32, K35, K36, K38, K48 |
| P47384 | MG_144 | 30S ribosomal protein S15 | K4, K11, K36, K40, K47, K53, K82, K91 |
| P47385 | MG_145 | Translation initiation factor IF-3 | K3, K13, K18, K22, K32, K34, K45, K52, K58, K64, K74, K75, K96, K101, K103, K113, K135 |
| P47386 | MG_146 | Acetate kinase | K6, K23, K73, K119, K136, K149, K152, K158, K164, K213, K221, K225, K237, K244, K259, K265, K290, K298, K302, K318, K326, K329, K333, K357, K361, K362, K364, K369, K373, K387, K393, K397, K398, K405, K411, K415, K418 |
| P47387 | MG_147 | Phosphate acetyltransferase | K4, K17, K18, K20, K29, K38, K43, K53, K63, K67, K79, K90, K99, K102, K111, K129, K133, K156, K161, K166 |
| P47388 | MG_148 | 50S ribosomal protein L35 | K3, K6, K9, K25, K37, K43, K57, K59, K63, K67, K72, K79, K82, K90, K95, K106, K108, K110, K114, K115, K133, K136, K137, K138 |
| P47390 | MG_149 | Pyruvate dehydrogenase E1 component subunit alpha | K3, K5, K52, K177, K184, K306, K374 |
| P47391 | MG_150 | Pyruvate dehydrogenase E1 component subunit beta | K38, K74, K129, K167, K314, K331, K347, K369, K374 |
| P47393 | MG_151 | Dihydrolipoyllysine-residue acetyltransferase component of pyruvate dehydrogenase complex | K4, K170, K182 |
| P47394 | MG_152 | Lipoamide-containing 2-oxo-acid dehydrogenase | K3, K6, K25, K40, K69, K99, K117, K133, K141, K142, K156, K196, K216, K228, K284, K309, K325, K330, K340, K353, K414, K458, K460, K478 |
| P47396 | MG_153 | Uncharacterized protein MG_153 | K692, K694, K706, K714, K730, K734, K750, K768, K785 |
| P47397 | MG_154 | Transcription elongation factor NusA | K10, K16, K19, K24, K28, K36, K42, K49, K63, K101, K147, K266, K360 |
| P47398 | MG_155 | Ribosome-binding factor A | K8, K25, K30, K41, K42, K46, K103, K116, K123 |
| P47399 | MG_156 | Leucine--tRNA ligase | K8, K82, K635, K671, K820 |
| P47400 | MG_157 | DNA polymerase III, alpha chain | K197, K215, K270, K309, K348, K454, K748, K879, K891, K914 |
| P47401 | MG_158 | tRNA-dihydrouridine(20/20a) synthase | K27, K73, K135, K215 |
| P47402 | MG_159 | GTP-binding protein TypA | K88, K118, K211, K225, K393, K573, K721, K732 |
| P47403 | MG_160 | Cysteine desulfurase | K13, K43, K162, K379, K383, K387, K574 |
| P47404 | MG_161 | GTP-binding protein EngA | K85, K691 |
| P47405 | MG_162 | DNA-directed RNA polymerase, delta subunit | K24, K25, K31, K45, K52, K54, K55, K67, K75, K92, K98, K101, K268, K272, K275, K305, K316 |
| P47406 | MG_163 | 50S ribosomal protein L3 | K6, K233, K268 |
| P47407 | MG_164 | 50S ribosomal protein L4 | K124, K136, K139 |
| P47408 | MG_166 | 50S ribosomal protein L23 | K197, K587 |
| P47409 | MG_167 | 50S ribosomal protein L22 | K19, K24, K107, K202, K426, K687 |
| P47410 | MG_169 | 30S ribosomal protein S3 | K4, K6, K11, K16, K23, K27, K31, K110, K112, K114, K136, K139, K147, K213, K229, K256, K299, K333, K353 |
| P47411 | MG_170 | 50S ribosomal protein L29 | K462, K496 |
| P47412 | MG_171 | 30S ribosomal protein S19 | K69, K96, K367, K572, K604, K642, K686 |
| P47413 | MG_172 | 50S ribosomal protein L14 | K249, K348, K365, K572 |
| P47414 | MG_173 | 50S ribosomal protein L24 | K8, K22, K25, K39, K45, K58, K61, K71, K85 |
| P47415 | MG_174 | 50S ribosomal protein L5 | K142, K229, K287, K300, K345, K446 |
| P47416 | MG_175 | 50S ribosomal protein L6 | K6, K42, K45, K80, K129, K200, K201, K223, K257 |
| P47417 | MG_176 | 30S ribosomal protein S5 | K27, K67, K148, K408 |
| P47418 | MG_177 | 50S ribosomal protein L18 | K56, K114, K554 |
| P47419 | MG_178 | 50S ribosomal protein L15 | K3, K6, K102, K150, K178, K292 |
| P47420 | MG_179 | 50S ribosomal protein L30 | K56, K75, K82, K106, K146, K178, K191, K194 |
| P47421 | MG_180 | Protein translocase subunit SecY | K182, K202, K204 |
| P47422 | MG_181 | Translation initiation factor IF-1 | K27, K36, K42, K504 |
| P47423 | MG_182 | 30S ribosomal protein S13 | K3, K4, K6, K7, K77, K101, K106, K112, K113, K115, K135, K140, K200, K232, K269, K287, K363, K475 |
| P47424 | MG_183 | DNA-directed RNA polymerase subunit alpha | K249, K294 |
| P47425 | MG_184 | 50S ribosomal protein L17 | K4, K13, K16, K17, K19, K22, K24, K34, K52, K58, K67, K78, K107, K118 |
| P47426 | MG_187 | ABC transporter ATP-binding protein | K184, K261 |
| P47429 | MG_189 | Probable oligoendopeptidase F | K15, K16, K22, K23, K24, K29, K30, K35, K47, K55, K62, K65, K82, K91, K94, K95, K98, K103, K119, K130, K134, K173, K178 |
| P47433 | MG_191 | PTS system, glucose-specific IIBC component | K6, K7, K9, K15, K18, K23, K24, K35, K39, K40, K41, K42, K46, K65, K81, K85, K112, K129, K132, K137, K139, K142, K143, K152, K163, K167, K220, K223, K230, K232, K237, K245, K256 |
| P47436 | MG_192 | Pyruvate dehydrogenase complex, E2 component | K81, K391, K695, K781, K792 |
| P47437 | MG_193 | Protein translocase subunit SecA | K98, K117, K137, K237, K304, K353, K501, K522, K551, K651, K769, K808, K863, K1095, K1266, K1330, K1342, K1405, K1501, K1568, K1574, K1700, K1704, K1693 |
| P47438 | MG_197 | Excinuclease ABC subunit B | K21, K50, K154, K197, K307 |
| P47389 | MG_199 | Oligopeptide ABC transporter, periplasmic oligopeptide-binding protein | K137 |
| P47393 | MG_200 | Oligopeptide transport system substrate-binding protein | K100, K111, K119, K125 |
| P47439 | MG_202 | 50S ribosomal protein L1 | K25, K76, K111, K143, K157 |
| P47440 | MG_205 | Elongation factor G | K3, K14, K17, K22, K27, K43, K50, K52, K60, K82, K123, K133, K151, K163, K204, K226, K239, K259, K262, K288, K299, K312, K325, K366, K373, K385, K519, K531, K598, K601, K612, K619, K626, K628, K664, K669, K694 |
| P47441 | MG_207 | Single-stranded DNA-binding protein | K2, K3, K16, K20, K22, K51, K73, K165 |
| P47442 | MG_208 | 30S ribosomal protein S18 | K2, K5, K10, K16, K24, K35, K38, K40, K41, K42, K49, K55, K62, K63, K72 |
| P47443 | MG_210 | 50S ribosomal protein L9 | K52, K278, K519, K546, K556, K568, K630, K680 |
| P47444 | MG_211 | Replicative DNA helicase | K23, K147, K231, K31, K44 |
| P47445 | MG_212 | Asparagine--tRNA ligase | K10, K11, K28, K32, K52, K71, K124, K130, K139, K141, K216, K221, K256, K292, K428 |
| P47446 | MG_214 | DNA-directed RNA polymerase, delta subunit | K5, K11, K12, K14, K15, K19, K24, K25, K31, K45, K52, K54, K55, K67, K75, K92, K98, K101, K268, K272, K275, K285, K305, K316 |
| P47447 | MG_215 | Ribonucleoside-diphosphate reductase subunit alpha | K3, K6, K8, K9, K23, K33, K65, K70, K136, K154, K178, K205, K220, K240, K266, K308, K362, K393, K425, K455, K466, K502, K521 |
| P47448 | MG_216 | 50S ribosomal protein L31 | K16, K23, K71, K124 |
| P47449 | MG_217 | Glucose-6-phosphate isomerase | K5, K16, K52, K71, K177, K184, K234, K306, K309, K366, K393, K458, K471 |
| P47450 | MG_218 | Probable asparagine--tRNA ligase | K292 |
| P47451 | MG_219 | Putative elongation factor G | K221, K65 |
| P47453 | MG_220 | Replication protein DnaC homolog | K36, K37, K43, K45, K52, K58, K60, K61, K74, K81, K82 |
| P47455 | MG_221 | DNA topoisomerase 1 | K11, K36, K145 |
| P47456 | MG_222 | Glycerol-3-phosphate dehydrogenase [NAD(P)+] | K126, K174, K201, K221, K268, K328, K502, K530, K533, K92 |
| P47457 | MG_223 | Glycerol kinase | K3, K49, K96, K105, K187, K252, K306, K360, K381, K521 |
| P47458 | MG_225 | Thioredoxin | K8, K21, K36, K41, K42, K48, K49, K50, K57, K59, K63, K64, K65, K67, K68, K80, K82, K92, K94, K96, K98 |
| P47459 | MG_226 | Tryptophanyl-tRNA synthetase | K65, K117, K139, K140, K152, K164, K172, K29, K40, K66, K98 |
| P47460 | MG_227 | 23S rRNA (uracil-5-)-methyltransferase RumA | K5, K10, K15, K19, K22, K29, K38, K42, K52, K82, K93, K108, K121, K125, K155, K159, K172, K241, K285, K315, K339, K340, K376, K462, K759, K791 |
| P47461 | MG_228 | Uncharacterized protein MG_228 | K285, K492 |
| P47463 | MG_229 | Polyphosphate kinase | K13, K125, K146, K158 |
| P47464 | MG_230 | Uncharacterized protein MG_230 | K263, K285 |
| P47465 | MG_231 | Uncharacterized protein MG_231 | K77, K85 |
| P47466 | MG_232 | Uncharacterized protein MG_232 | K3, K131, K21, K24, K34, K43, K197, K666, K900 |
| P47467 | MG_233 | Putative sugar kinase | K8, K82 |
| P47468 | MG_234 | Uncharacterized protein MG_234 | K10, K15, K19, K22, K29, K38, K121, K125 |
| P47469 | MG_235 | Uncharacterized protein MG_235 | K5, K37, K52, K57, K91, K101 |
| P47470 | MG_236 | Uncharacterized protein MG_236 | K142, K156, K325 |
| P47471 | MG_237 | Uncharacterized protein MG_237 | K25, K40, K69, K99 |
| P47472 | MG_239 | Uncharacterized protein MG_239 | K133, K141, K216, K228, K284, K309, K325, K330, K340, K353 |
| P47473 | MG_240 | Uncharacterized protein MG_240 | K3, K4 |
| P47474 | MG_241 | Uncharacterized protein MG_241 | K347, K369 |
| P47476 | MG_243 | Ribonucleoside-diphosphate reductase subunit alpha | K38, K74, K167 |
| P47477 | MG_245 | Peptide deformylase | K5, K52, K263, K285, K306, K374 |
| P47478 | MG_246 | Glucose-6-phosphate isomerase | K5, K52, K177, K184, K306 |
| P47479 | MG_248 | Uncharacterized protein MG_248 | K57, K83, K243 |
| P47480 | MG_249 | Asparagine--tRNA ligase | K6, K292, K11, K226, K230, K263, K295, K38, K101, K133, K161, K171, K22, K126, K174, K201, K221, K268, K328, K502, K530, K533, K92 |
| P47481 | MG_252 | DNA topoisomerase 1 | K5, K11, K36, K145, K3, K49, K96, K105, K187, K252, K306, K360, K381, K521 |
| P47482 | MG_255 | Uncharacterized protein MG_255 | K8, K68, K65, K117, K30, K146, K241, K563, K207, K249, K559, K240, K4, K10, K28, K35, K54, K121, K137, K140, K150, K215, K217, K348, K120 |
| P47483 | MG_261 | Thioredoxin | K215, K263, K945, K530, K536, K543, K549, K592, K666, K806, K875, K971, K1006, K536, K549, K30, K31, K72, K93, K96, K110, K112, K114, K138, K170, K238, K439 |
| P47484 | MG_262 | Uncharacterized protein MG_262 | K10, K273, K72, K273, K825, K872, K993, K1058, K1060 |
| P47486 | MG_263 | Uncharacterized protein MG_263 | K256, K318, K996 |
| P47487 | MG_264 | Uncharacterized protein MG_264 | K37, K100, K138, K16, K19, K48, K173 |
| P47488 | MG_265 | Uncharacterized protein MG_265 | K19, K106, K130, K150, K164, K173, K16, K48, K55, K60 |
| P47491 | MG_266 | Uncharacterized protein MG_266 | K28, K169, K191, K286, K323, K368, K417, K561, K684, K191, K266, K353, K123, K366, K50, K285, K67 |
| P47492 | MG_267 | Polyphosphate kinase | K86, K91, K125 |
| P47493 | MG_268 | Uncharacterized protein MG_268 | K17, K101, K37, K101 |
| P47494 | MG_269 | Uncharacterized protein MG_269 | K312, K285, K492 |
| P47495 | MG_270 | Uncharacterized protein MG_270 | K13, K158, K263, K285, K77, K85 |
| P47496 | MG_271 | Uncharacterized protein MG_271 | K3, K6, K8, K9, K23, K33, K65, K70, K136, K178, K205, K220, K240, K266, K308, K362, K393, K425, K455, K466, K502, K521 |
| P47498 | MG_273 | Putative hemolysin-like protein | K3, K67, K108, K111, K121, K140, K236, K342 |
| P47499 | MG_274 | Cytadherence-accessory protein HMW3 | K4, K13, K122 |
| P47500 | MG_275 | Uncharacterized protein MG_275 | K98, K162, K272, K292, K363 |
| P47505 | MG_276 | Uncharacterized protein MG_276 | K6, K68, K945 |
| P47506 | MG_279 | Putative transport system permease protein | K3, K65, K81, K83, K87, K96, K117, K121, K174, K201, K268, K308 |
| P47507 | MG_280 | Probable thymidylate synthase | K28, K46, K88, K134, K143, K219 |
| P47508 | MG_282 | Putative dihydrofolate reductase | K60, K66, K74, K267 |
| P47510 | MG_285 | Ribonucleoside-diphosphate reductase subunit beta | K62, K84 |
| P47512 | MG_287 | Ribonucleoside-diphosphate reductase activating protein | K5, K38, K52, K285, K290, K306, K308, K310, K567, K574 |
| P47513 | MG_288 | Ribonucleoside-diphosphate reductase subunit alpha | K91, K112, K166, K279, K504, K537, K541, K565 |
| P47514 | MG_289 | Uncharacterized protein MG_289 | K310, K504, K537 |
| P47515 | MG_290 | 50S ribosomal protein L21 | K5, K142, K331, K486, K493, K528, K549, K820, K873, K876, K345 |
| P47516 | MG_291 | Uncharacterized protein MG_291 | K4, K56 |
| P47518 | MG_292 | Chaperone protein Tig | K73, K224, K349, K416, K440 |
| P47520 | MG_293 | Lon protease | K48, K536, K549, K530, K536, K592, K666, K806, K875, K971, K1006 |
| P47524 | MG_294 | Mpn294 | K30, K31, K72, K93, K96, K110, K112, K114, K138, K170, K238, K439 |
| P47525 | MG_295 | Uncharacterized protein MG_295 | K10, K273, K72, K273, K825, K872, K993, K1058, K1060 |
| P47529 | MG_296 | Uncharacterized protein MG_296 | K256, K318, K996 |
| P47530 | MG_297 | Uncharacterized protein MG_297 | K37, K100, K138 |
| P47532 | MG_298 | MG298 | K16, K19, K48, K173 |
| P47534 | MG_299 | MG299 | K16, K19, K48, K55, K60, K106, K130, K150, K164, K173 |
| P47537 | MG_300 | MG300 | K28, K169, K191, K286, K323, K368, K417, K561, K684, K191, K266, K353 |
| P47538 | MG_302 | MG302 | K50, K123, K366 |
| P47539 | MG_303 | PepF protein homolog | K285, K67, K86, K91, K125, K17, K101, K37, K101 |
| P47540 | MG_304 | MG304 | K312, K285, K492, K13, K125, K146, K158, K263, K285, K77, K85 |
| P47541 | MG_305 | MG305 | K3, K131, K8, K82, K10, K15, K19, K22, K29, K38, K121, K125 |
| P47542 | MG_306 | MG306 | K5, K37, K52, K57, K91, K101, K285, K312, K142, K156, K325 |
| P47543 | MG_308 | Uncharacterized protein MG_308 | K25, K40, K69, K99, K133, K141, K216, K228, K284, K309, K325, K330, K340, K353, K3, K4 |
| P47546 | MG_309 | HMW1 protein | K347, K369, K38, K74, K167, K5, K52, K263, K285, K306, K374, K5, K52, K177, K184, K306, K57, K83, K243, K6, K292 |
| P47547 | MG_310 | Hmw2 | K11, K22, K38, K101, K133, K161, K171, K226, K230, K263, K295, K5, K11, K36, K145 |
| P47548 | MG_312 | Putative ATP-dependent protease ATPase subunit | K92, K126, K174, K201, K221, K268, K328, K502, K530, K533 |
| P47553 | MG_313 | Uncharacterized protein MG_313 | K3, K49, K96, K105, K187, K252, K306, K360, K381, K521 |
| P47561 | MG_314 | MraZ protein | K8, K68 |
| P47563 | MG_315 | MraW protein | K2, K5, K10, K13, K27, K28, K37, K43, K44, K60, K65 |
| P47565 | MG_316 | Thymidylate synthase | K12, K24, K47, K54, K62, K71, K72, K84, K96, K98 |
| P47566 | MG_317 | MG317 | K6, K22, K42, K44, K55, K62, K63, K64, K75, K90, K98, K102, K105, K124, K125, K126, K130, K135 |
| P47567 | MG_318 | Dihydrofolate reductase | K15, K16, K26, K28, K34 |
| P47568 | MG_320 | Ribonucleoside-diphosphate reductase subunit beta | K12, K17, K19, K22, K37, K42, K45, K52, K62, K63, K68, K88, K91, K93, K96, K105, K106, K111, K115, K123, K130, K131, K132, K136, K147, K148, K152, K154, K159 |
| P47569 | MG_321 | Ribonucleoside-diphosphate reductase activating protein | K2, K10, K11, K23, K27, K37, K50 |
| P47570 | MG_322 | Ribonucleoside-diphosphate reductase subunit alpha | K4, K11, K14, K16, K27, K43, K57, K77, K86, K87, K93, K107 |
| P47571 | MG_323 | 50S ribosomal protein L21 | K4, K5, K6, K8, K10, K34, K40 |
| P47572 | MG_324 | Uncharacterized protein MG_324 | K10, K13, K16, K18, K21, K23, K24, K32 |
| P47573 | MG_325 | Uncharacterized protein MG_325 | K4, K8, K12, K14, K24, K25, K27, K35, K37 |
| P47574 | MG_328 | Chaperone protein Tig | K3, K20, K24, K47, K49 |
| P47575 | MG_329 | ATP-dependent Lon protease | K3, K9, K13, K24, K26, K32, K51, K82, K92 |
| P47576 | MG_330 | ATP-dependent Lon protease | K6, K8, K12, K13, K20, K26, K32, K33, K35, K38, K41, K42, K52, K53, K9, K25, K82, K90, K135, K203 |
| P47577 | MG_331 | Uncharacterized protein MG_331 | K3, K5, K13, K14, K27, K40, K41, K42, K49, K55, K62, K63, K72 |
| P47579 | MG_332 | Uncharacterized protein MG_332 | K10, K11, K28, K35, K54, K120, K240 |
| P47582 | MG_336 | Uncharacterized protein MG_336 | K3, K8, K10, K12, K13, K16, K24, K25, K32, K37, K39, K40, K41, K42, K43, K50, K52, K55, K59, K63, K68, K71, K74, K75, K76, K77, K78, K80, K82, K86, K88, K89, K90, K95, K96, K97, K102, K104, K105, K106, K112, K114, K120, K121, K123, K124 |
| P47583 | MG_337 | Uncharacterized protein MG_337 | K2, K3, K4, K6, K8, K9, K10, K12, K13, K20, K23, K24, K25, K26, K27, K28, K32, K34, K35, K37, K38, K41, K42, K43, K44, K46, K47, K49, K50, K52, K53, K55, K56, K58, K60, K61, K63, K64, K65, K67, K69, K71, K72, K74, K75, K76, K77, K78, K80, K81, K82, K84, K86, K87, K88, K89, K90, K91, K92, K93, K95, K96, K97, K98, K101, K102, K103, K104, K105, K106, K107, K108, K110, K111, K112, K114, K115, K116, K117, K119, K120, K121, K123, K124, K125, K126, K128, K129, K132, K133, K134, K135, K136, K137, K138, K140, K141, K142 |
| P47584 | MG_348 | RNA polymerase sigma factor RpoD | K2, K11, K12, K14, K15, K22, K27, K43, K56, K63, K82, K97, K100, K123, K130, K165, K279 |
| P47585 | MG_349 | DNA primase | K21, K24, K28, K35, K38, K48, K53, K65, K84, K96, K100, K103, K115, K120, K124, K128, K132, K140, K155, K157, K162, K164, K172, K220, K225, K247, K259, K269, K314, K336, K346 |
| P47587 | MG_352 | Glycyl-tRNA synthetase | K10, K26, K28, K36, K38, K41, K52, K57, K86, K92, K98, K107, K108, K122 |
| P47588 | MG_353 | Uncharacterized protein MG_353 | K8, K10, K17, K19, K22, K43, K45, K49, K63, K64, K75, K92, K110, K150, K159, K224, K234, K255, K260, K278, K302, K320, K330, K346, K388 |
| P47589 | MG_354 | 50S ribosomal protein L31, type B | K2, K18, K66, K106, K135, K141 |
| P47591 | MG_359 | Peptide chain release factor 1 | K21, K28, K30, K45, K76, K87, K93, K99, K101, K113, K115, K117, K132, K133, K144, K154, K159, K161, K173 |
| P47592 | MG_360 | Uncharacterized protein MG_360 | K353, K630, K701, K822, K848 |
| P47593 | MG_361 | Uncharacterized protein MG_361 | K131, K223, K225, K253, K259, K261, K303, K312, K333, K359, K363, K428 |
| P47595 | MG_362 | Uncharacterized protein MG_362 | K187, K220, K225, K253, K290, K11, K78, K104, K3, K20, K36, K42, K49, K55 |
| P47596 | MG_365 | Uncharacterized protein MG_365 | K23, K34, K155, K167, K192, K205, K213 |
| P47597 | MG_366 | Uncharacterized protein MG_366 | K26, K44, K52, K76, K132, K176, K23, K62 |
| P47599 | MG_368 | DNA-directed RNA polymerase subunit beta' | K14, K35, K118, K184, K352, K760, K960, K14, K65, K204, K244, K290, K21, K10, K52, K27, K37, K40, K115, K122, K136, K179, K270, K344, K345 |
| P47603 | MG_372 | Uncharacterized protein MG_372 | K5, K7, K9, K21, K42, K49, K74, K178, K232, K234, K241 |
| P47604 | MG_374 | Uncharacterized protein MG_374 | K26, K173 |
| P47605 | MG_375 | Uncharacterized protein MG_375 | K126, K229, K114 |
| P47606 | MG_376 | Uncharacterized protein MG_376 | K116, K128 |
| P47607 | MG_377 | Uncharacterized protein MG_377 | K4, K11, K22, K34, K42, K45, K76, K155, K101, K105, K132, K152, K194, K238, K248, K252, K354 |
| P47609 | MG_379 | DNA polymerase I | K6, K21, K77, K181, K203, K204, K359, K23, K119, K136, K164, K213, K290, K329, K152, K3, K29, K31 |
| P47610 | MG_381 | 50S ribosomal protein L10 | K21, K26, K31, K41, K42, K47, K56, K57, K59, K63, K68, K69, K75, K79 |
| P47612 | MG_384 | Leucyl-tRNA synthetase | K2, K11, K14, K15, K19, K20, K24, K25, K26, K34, K40, K41, K51, K55, K59, K66, K77, K86, K87, K96 |
| P47613 | MG_386 | Uncharacterized protein MG_386 | K14, K34, K38, K51, K45, K50 |
| P47614 | MG_387 | Uncharacterized protein MG_387 | K5, K34, K47, K53, K65, K102, K116, K136, K356 |
| P47615 | MG_388 | Lipoyl-protein ligase A | K356, K364, K388, K425, K446, K460, K472, K511, K643, K28, K33, K51, K59, K72, K105, K118, K159, K229, K232, K463 |
| P47616 | MG_389 | Dihydrolipoamide dehydrogenase | K142, K20, K129, K226, K229, K230, K270, K296, K302, K325, K333, K347 |
| P47617 | MG_390 | Dihydrolipoamide acetyltransferase | K17, K18, K57, K62, K63, K99, K130, K150 |
| P47618 | MG_391 | Pyruvate dehydrogenase E1 component subunit beta | K9, K82, K203, K228 |
| P47619 | MG_392 | Pyruvate dehydrogenase E1 component subunit alpha | K5, K13, K14, K27, K40, K80 |
| P47620 | MG_393 | NADH oxidase | K2, K3, K6, K9, K12, K13, K20, K23, K24, K25, K27, K28, K32, K34, K35, K37, K38, K41, K42, K43, K44, K46, K47, K49, K50, K52, K53, K55, K56, K58, K60, K61, K63, K64, K65, K67, K69, K71, K72, K74, K75, K76, K77, K78, K80, K81, K82, K84, K86, K87, K88, K89, K90, K91, K92, K93, K95, K96, K97, K98, K101, K102, K103, K104, K105, K106, K107, K108, K110, K111, K112, K114, K115, K116, K117, K119, K120, K121, K123, K124, K125, K126, K128, K129, K132, K133, K134, K135, K136, K137, K138, K140, K141, K142 |
| P47621 | MG_394 | Bifunctional protein SpoT | K69, K148, K191, K201, K230, K341 |
| P47622 | MG_395 | Transcription elongation factor GreA | K73, K176, K182, K267, K364, K432 |
| P47623 | MG_396 | Uncharacterized protein MG_396 | K19, K93, K101, K142, K145, K171, K252, K134, K177 |
| P47624 | MG_397 | Prolyl-tRNA synthetase | K6, K22, K29, K37, K38, K42, K67, K126, K295 |
| P47625 | MG_398 | Alanyl-tRNA synthetase | K4, K18, K88, K92, K125, K134, K156, K254, K255, K282, K310 |
| P47627 | MG_399 | tRNA-specific-2-thiouridylase MnmA | K274, K406, K3, K29, K38, K54, K83, K501 |
| P47631 | MG_401 | Signal recognition particle protein | K6, K8, K698, K8, K242, K3, K221, K83, K87, K96, K117, K121, K174, K201, K268, K30, K134 |
| P47632 | MG_402 | Uncharacterized protein MG_402 | K30, K46, K88, K28, K143, K219, K60, K66, K74, K74, K267 |
| P47633 | MG_410 | Protein-export protein SecG | K62, K84, K5, K38, K52, K285, K290, K306, K567, K574, K308,K310 |
| P47634 | MG_419 | Phosphate acetyltransferase | K284, K91, K112, K166, K279, K504, K537, K541, K565, K310, K504, K537 |
| P47635 | MG_422 | Phosphoglycerate kinase | K5, K142, K331, K486, K493, K528, K549, K820, K873, K876, K345 |
| P47636 | MG_425 | Glyceraldehyde-3-phosphate dehydrogenase | K4, K56 |
| P47637 | MG_426 | DnaK protein | K48, K73, K224, K349, K416, K440 |
| P47638 | MG_428 | Adhesin P1 | K536, K549, K530, K536, K592, K666, K806, K875, K971, K1006 |
| P47639 | MG_429 | HMW3 protein | K30, K31, K72, K93, K96, K110, K112, K114, K138, K170, K238, K439 |
| P47640 | MG_430 | Uncharacterized protein MG_430 | K10, K273, K72, K273, K825, K872, K993, K1058, K1060, K256, K318, K996 |
| P47641 | MG_434 | Uncharacterized protein MG_434 | K37, K100, K138, K16, K19, K48, K173, K19, K60, K106, K130, K150, K164, K173, K16, K48, K55 |
| P47642 | MG_447 | Endopeptidase P \| Endopeptidase P-like protein | K28, K169, K191, K286, K323, K368, K417, K561, K684 |
| P47643 | MG_452 | 50S ribosomal protein L33-2 | K191, K266, K353, K50, K123, K366, K285, K67 |
| P47645 | MG_453 | Uncharacterized protein MG_453 | K86, K91, K125 |
| P47647 | MG_461 | Cytidylate kinase | K17, K101, K37, K101, K312, K285, K492, K13, K125, K146, K158, K263, K285, K77, K85 |
| P47648 | MG_470 | Putative NADH-dependent flavin oxidoreductase | K3, K131, K8, K82 |
| P47649 | MG_471 | Valine--tRNA ligase | K10, K15, K19, K22, K29, K38, K121, K125, K5, K37, K52, K57, K91, K101, K285, K312 |
| P47650 | MG_472 | Uncharacterized protein MG_472 | K142, K156, K325 |
| P47655 | MG_474 | Putative phosphosugar isomerase | K25, K40, K69, K99, K133, K141, K216, K228, K284, K309, K325, K330, K340, K353, K3, K4, K347, K369 |
| P47656 | MG_475 | Putative sugar kinase \| ATP-grasp domain-containing protein \| Nucleotide-diphospho-sugar transferases | K38, K74, K167 |
| P47657 | MG_476 | Cytidylate kinase | K5, K52, K263, K285, K306, K374, K5, K52, K177, K184, K306 |
| P47658 | MG_478 | Probable FAD-dependent pyridine nucleotide-disulfide oxidoreductase | K6, K57, K83, K226 |
| P47660 | MG_479 | Valine--tRNA ligase | K668, K315, K692 |
| P47661 | MG_480 | Putative phosphosugar isomerase | K419, K606 |
| P47662 | MG_492 | DNA-directed RNA polymerase subunit beta | K23, K9, K70, K81, K308, K161, K196, K200, K206, K298, K304, K342, K13, K28, K36, K50, K65, K67, K111, K116, K120, K194, K197, K9, K16, K27, K28, K45, K80, K98, K112, K133, K205, K249 |
| P47663 | MG_495 | DNA-directed RNA polymerase subunit beta' | K16, K107, K110, K127, K368, K372 |
| P47666 | MG_499 | Uncharacterized protein MG_499 | K6, K24, K462, K496 |
| P47667 | MG_502 | Uncharacterized protein MG_502 | K4, K19, K24, K107, K202, K426, K687 |
| P47668 | MG_506 | Uncharacterized protein MG_506 | K4, K6, K11, K16, K23, K27, K31, K110, K112, K114, K136, K139, K147, K213, K229, K256, K299, K333, K353, K69, K142, K202, K426, K686, K69, K96, K367, K572, K604, K642 |
| P47669 | MG_515 | Uncharacterized protein MG_515 | K249, K348, K365, K572, K8, K22, K25, K39, K45, K58, K61, K71, K85 |
| P47670 | MG_516 | Uncharacterized protein MG_516 | K142, K229, K287, K300, K345, K446, K6, K42, K45, K80, K129, K200, K201, K223, K257 |
| P47672 | MG_517 | Protein P1 | K27, K67, K148, K408, K56, K114, K554 |
| P47673 | MG_518 | Uncharacterized protein MG_518 | K3, K6, K102, K150, K178, K292, K56, K75, K82, K106, K146, K178, K191, K194, K182, K202, K204 |
| P47678 | MG_521 | Uncharacterized protein MG_521 | K504 |
| P47681 | MG_526 | Inorganic pyrophosphatase | K4, K6, K7, K77, K101, K106, K112, K113, K115, K135, K140, K200, K232, K269, K287, K363, K475 |
| P47682 | MG_528 | Uncharacterized protein MG_528 | K249, K294 |
| P47683 | MG_529 | Uncharacterized protein MG_529 | K107 |
| P47684 | MG_530 | ATP-dependent Clp protease ATP-binding subunit ClpB | K184, K261 |
| P47685 | MG_531 | Acetate kinase | K15, K16, K22, K23, K24, K29, K30, K35, K47, K55, K62, K65, K82, K91, K94, K95, K98, K103, K119, K130, K134, K173, K178 |
| P47686 | MG_533 | 50S ribosomal protein L10 | K1214, K1708, K6, K7, K9, K15, K18, K23, K24, K35, K39, K40, K41, K42, K46, K65, K81, K85, K112, K129, K132, K137, K139, K142, K143, K152, K163, K167, K220, K223, K230, K232, K237, K245, K256 |
| P47688 | MG_534 | 50S ribosomal protein L7/L12 | K391, K695, K781, K792, K81, K98, K117, K501, K522, K551, K769, K863, K1095, K1266, K1330, K1342, K1405, K1501, K1568, K1574, K1693, K1700, K1704 |
| P47691 | MG_535 | 30S ribosomal protein S20 | K21, K50, K154, K197, K307, K119, K137, K100, K111, K125, K25, K76, K111, K143, K157, K3, K14, K17, K22, K27, K43, K50, K52, K60, K82, K123, K133, K151, K163, K204, K226, K239, K259, K262, K288, K299, K312, K325, K366, K373, K385, K519, K531, K598, K601, K612, K619, K626, K628, K664, K669, K694, K20, K165 |
| P47692 | MG_538 | Ribonuclease III | K52, K278, K519, K546, K556, K568, K630, K680, K31, K44, K23, K147 |
| P47693 | MG_539 | Acyltransferase | K28, K333, K12, K285, K154 |
| P47694 | MG_541 | Uncharacterized protein MG_541 | K107, K120, K130, K147, K213, K225, K238, K35, K54, K55, K772 |
| P47695 | MG_542 | Uncharacterized protein MG_542 | K16, K71, K234, K393, K292, K11, K22, K38, K101, K133, K161, K171, K226, K230, K263, K295, K5, K11, K36, K145, K92, K126, K174, K201, K221, K268, K328, K502, K530, K533 |
| P47696 | MG_543 | Threonyl-tRNA synthetase | K3, K49, K96, K105, K187, K252, K306, K360, K381, K521, K8, K68 |
| P47698 | MG_544 | Arginine--tRNA ligase | K65, K117, K30, K146, K241, K563, K207, K249, K559, K240, K4, K10, K28, K35, K54, K121, K137, K140, K150, K215, K217, K348, K120, K215, K263, K945 |
| P47699 | MG_545 | GTP-binding protein RsmG | K530, K536, K543, K549, K592, K666, K806, K875, K971, K1006, K536, K549, K30, K31, K72, K93, K96, K110, K112, K114, K138, K170, K238, K439, K10, K273 |
| P47700 | MG_546 | GTP-binding protein Obg | K72, K273, K825, K872, K993, K1058, K1060, K256, K318, K996, K37, K100, K138, K16, K19, K48, K173, K19, K60, K106, K130, K150, K164, K173, K16, K48, K55, K28, K169, K191, K286, K323, K368, K417, K561, K684, K191 |
| P47702 | MG_547 | Peptide deformylase-like protein | K266, K353, K123, K366, K50 |
| P47704 | MG_553 | Peptidyl-prolyl cis-trans isomerase A | K285 |
| P47705 | MG_555 | 60 kDa chaperonin | K67, K86, K91, K125 |
| P47706 | MG_556 | 10 kDa chaperonin | K17, K101, K37, K101, K312, K285, K492, K13, K125, K146, K158, K263, K285 |
| P52271 | MG_561 | ATP synthase subunit alpha | K77, K85 |
| P55750 | MG_562 | ATP synthase subunit gamma | K3, K131, K8, K82, K10, K15, K19, K22, K29, K38, K121, K125 |
| P56723 | MG_563 | ATP synthase subunit beta | K5, K37, K52, K57, K91, K101, K285, K312, K142, K156, K325 |
| P57085 | MG_564 | ATP synthase F0 sector subunit b' | K25, K40, K69, K99, K133, K141, K216, K228, K284, K309, K325, K330, K340, K353 |
| Q2MHT0 | MG_565 | ATP synthase F0 sector subunit a | K3, K4 |
| Q49310 | MG_566 | Enolase | K347, K369 |
| Q49329 | MG_567 | Putative phospholipid/glycerol acyltransferase | K38, K74, K167 |
| Q49396 | MG_568 | PTS system, IIBC component | K5, K52, K263, K285, K306, K374, K5, K52, K177, K184, K306, K6, K57, K83, K243, K292 |
| Q49397 | MG_572 | Uncharacterized protein MG_572 | K668 |
| Q49398 | MG_573 | 60 kDa chaperonin | K315, K692 |
| Q49399 | MG_574 | 10 kDa chaperonin | K419, K606, K23, K9 |
| Q49400 | MG_575 | 30S ribosomal protein S9 | K70, K81, K308 |
| Q49401 | MG_576 | 30S ribosomal protein S9 | K161, K196, K200, K206, K298, K304, K342 |
| Q49402 | MG_577 | Excinuclease ABC subunit A | K13, K28, K36, K50, K65, K67, K111, K116, K120, K194, K197 |
| Q49404 | MG_578 | Uncharacterized protein MG_578 | K9, K16, K27, K28, K45, K80, K98, K112, K133, K205, K249 |
| Q49405 | MG_584 | Uncharacterized protein MG_584 | K16, K107, K110, K127, K368, K372 |
| Q49406 | MG_588 | Uncharacterized protein MG_588 | K24, K45, K102, K231, K275, K368, K462, K496 |
| Q49407 | MG_590 | Uncharacterized protein MG_590 | K4, K6, K11, K16, K19, K23, K24, K27, K31, K107, K110, K112, K114, K136, K139, K147, K213, K229, K256, K299, K333, K353, K687 |
| Q49408 | MG_591 | Uncharacterized protein MG_591 | K69, K96, K142, K202, K367, K426, K572, K604, K642, K686 |
| Q49412 | MG_592 | Uncharacterized protein MG_592 | K249, K348, K365, K572, K8, K22, K25, K39, K45, K58, K61, K71, K85 |
| Q49413 | MG_595 | Uncharacterized protein MG_595 | K142, K229, K287, K300, K345, K446, K6, K42, K45, K80, K129, K200, K201, K223, K257 |
| Q49415 | MG_596 | Uncharacterized protein MG_596 | K27, K67, K148, K408, K56, K114, K554 |
| Q49416 | MG_598 | Uncharacterized protein MG_598 | K3, K6, K102, K150, K178, K292, K56, K75, K82, K106, K146, K178, K191, K194 |
| Q49417 | MG_599 | Uncharacterized protein MG_599 | K182, K202, K204 |
| P47683 | MG_600 | Uncharacterized protein MG_600 | K273, K72, K825, K872, K993, K1058, K1060 |
| P47687 | MG_601 | Uncharacterized protein MG_601 | K28, K191, K286, K323, K368, K417, K561, K684, K169 |
| P47688 | MG_602 | Uncharacterized protein MG_602 | K191 |
| P47690 | MG_606 | Uncharacterized protein MG_606 | K123, K366, K266, K353 |
| P47692 | MG_608 | Uncharacterized protein MG_608 | K50, K285, K67 |
| P47693 | MG_609 | PTS system, N-acetylglucosamine-specific IIBCA component | K86, K91, K125 |
| P47694 | MG_610 | PTS system, N-acetylglucosamine-specific IIBCA component | K17, K101 |
| P47695 | MG_616 | 30S ribosomal protein S9 | K37, K101 |
| P47696 | MG_619 | Excinuclease ABC subunit A | K285, K312, K492 |
| P47698 | MG_621 | Uncharacterized protein MG_621 | K13, K125, K146, K158, K263, K285, K77, K85, K3, K131, K8, K82 |
| P47699 | MG_622 | Ribosomal protein S21 homolog | K10, K15, K19, K22, K29, K38, K121, K125, K159, K172, K241, K285, K315, K339, K340, K376 |
| P47700 | MG_625 | Uncharacterized protein MG_625 | K5, K37, K52, K57, K91, K101, K285, K312, K142, K156, K325 |
| P47702 | MG_627 | Phosphoglyceromutase family protein | K25, K40, K69, K99 |
| P47704 | MG_628 | Glyceraldehyde-3-phosphate dehydrogenase | K133, K141, K216, K228, K284, K309, K325, K330, K340, K353, K3, K4 |
| P47705 | MG_629 | Triosephosphate isomerase | K347, K369 |
| P47706 | MG_631 | Elongation factor Ts | K38, K74, K167, K5, K52, K263, K285, K306, K374 |
| P47706 | MG_636 | Ribosome-recycling factor | K5, K52, K177, K184, K306 |
| Q57081 | MG_638 | Uncharacterized protein MG_638 | K6, K22, K29, K37, K38, K42, K67, K126, K295, K4, K18, K88, K92, K125, K134, K156, K254, K255, K282, K310, K274, K406, K3, K29, K38, K54, K83, K501 |
| Q9ZB70 | MG_640 | Uncharacterized protein MG_640 | K6, K8, K698, K8, K242 |
| Q9ZB72 | MG_642 | Uncharacterized protein MG_642 | K3, K221, K83, K87, K96, K117, K121, K174, K201, K268 |
| Q9ZB73 | MG_643 | Uncharacterized protein MG_643 | K30, K134, K30, K46, K88, K28, K143, K219 |
| Q9ZB76 | MG_644 | Uncharacterized protein MG_644 | K60, K66, K74, K74, K267 |
| Q9ZB78 | MG_645 | Uncharacterized protein MG_645 | K62, K84, K5, K38, K52, K285, K290, K306, K567, K574, K308,K310 |
| Q9ZB79 | MG_646 | Uncharacterized protein MG_646 | K284 |
