# Supplementary material for: Survival strategies of mycoplasmas: the critical role of post-translational modifications
Source: Front Cell Infect Microbiol. 2025 Nov 26;15:1688880. doi: 10.3389/fcimb.2025.1688880 (PMC12689956; doi:10.3389/fcimb.2025.1688880)
Supplement: Supplementary file 3 [file Table3.docx]

| **Supplementary Table 3. Identified Glycosylation Sites in Mycoplasmas** | | | |
| --- | --- | --- | --- |
| Accession | Systematic name | Protein name | Glycosylation Site (S) |
| AAC71620.2 | MG_219 | Chaperonin GroEL | N45 |
| AAC71566.1 | MG_164 | DNA-directed RNA polymerase subunit beta | N319 |
| AAC72471.1 | MG_450 | Elongation Factor Tu | S142 |
| AAC71307.1 | MG_014 | Elongation Factor G | S302 |
| AAC71523.1 | MG_121 | Glyceraldehyde-3-phosphate dehydrogenase | S107 |
| AAC71495.1 | MG_093 | Pyruvate dehydrogenase E1 component subunit beta | T134, E307 |
| AAC71582.1 | MG_180 | Acetate kinase | N10 |
| AAC71521.1 | MG_119 | Phosphate acetyltransferase | S102, D100 |
| AAC72448.1 | MG_427 | Hydroperoxide reductase | N123 |
| AAC71293.1 | MG_075 | Uncharacterized protein MG075 | S581 |
| AAC71503.1 | MG_281 | Uncharacterized protein MG281 [Protein M] | N202 |
| AAC71410.1 | MG_191 | Adhesin P1 [MgPa] | E407 |
| AVX54645.1 | MPN567 | EF-Tu | T159, Y161, E216 |
| AVX54671.1 | MPN309 | Dihydrolipoyl dehydrogenase | T14 |
| AVX54812.1 | MPN126 | L-lactate dehydrogenase | Y234 |
| AVX54801.1 | MPN140 | Uncharacterized lipoprotein | Q925 |
| AVX54673.1 | MPN307 | Acetate kinase | S178 |
| AVX54670.1 | MPN310 | Branched-chain alpha keto acid dehydrogenase | T14 |
| AVX54879.1 | MPN050 | Fatty acid binding protein | D8 |
| AVX54662.1 | MPN407 | Phosphopyruvate hydratase | T383 |
| AVX54718.1 | MPN225 | Transketolase | Y279 |
| AVX54783.1 | MPN154 | Fatty acid kinase subunit A | Y196 |
| SRX69133.1 | MPN047 | Glycerol ABC transporter | N169 |
| SRX69148.1 | MPN126 | L-lactate dehydrogenase | N123 |
| SRX69207.1 | MPN476 | DnaK | E564 |
| SRX68848.1 | MPN494 | ABC transporter | E479 |
| SRX68831.1 | MPN567 | EF-Tu | E216 |
| SRX69115.1 | MPN140 | Lipoprotein | Q925 |
| SRX68746.1 | MPN607 | Alanine dehydrogenase | S298 |
| SRX68910.1 | MPN309 | Dihydrolipoyl dehydrogenase | E11 |
| SRX71236.1 | MPN256 | Uncharacterized protein | T270 |
| SRX69114.1 | MPN141 | Lipoprotein | S35 |
| SRX68912.1 | MPN307 | Acetate kinase | T54 |
| SRX69241.1 | MPN004 | MOLPALP family lipoprotein | N680 |
| SRX68847.1 | MPN495 | Peptide ABC transporter ATP-binding protein | Y218 |
| SRX68730.1 | MPN627 | MurR/RpiR family transcriptional regulator | N107 |
